# A comparison of various imputation algorithms for missing data

**DOI:** 10.1371/journal.pone.0319784

**Published:** 2025-05-12

**Authors:** Jürgen Kampf, Iryna Dykun, Tienush Rassaf, Amir Abbas Mahabadi

**Affiliations:** Department of Cardiology and Vascular Medicine, University Hospital of Essen, Essen, Germany; University of Oviedo: Universidad de Oviedo, SPAIN

## Abstract

**Background:**

Many datasets in medicine and other branches of science are incomplete. In this article we compare various imputation algorithms for missing data.

**Objectives:**

We take the point of view that it has already been decided that the imputation should be carried out using multiple imputation by chained equation and the only decision left is that of a subroutine for the one-dimensional imputations. The subroutines to be compared are predictive mean matching, weighted predictive mean matching, sampling, classification or regression trees and random forests.

**Methods:**

We compare these subroutines on real data and on simulated data. We consider the estimation of expected values, variances and coefficients of linear regression models, logistic regression models and Cox regression models. As real data we use data of the survival times after the diagnosis of an obstructive coronary artery disease with systolic blood pressure, LDL, diabetes, smoking behavior and family history of premature heart diseases as variables for which values have to be imputed. While we are mainly interested in statistical properties like biases, mean squared errors or coverage probabilities of confidence intervals, we also have an eye on the computation time.

**Results:**

Weighted predictive mean matching had to be excluded from the statistical comparison due to its enormous computation time. Among the remaining algorithms, in most situations we tested, predictive mean matching performed best.

**Novelty:**

This is by far the largest comparison study for subroutines of multiple imputation by chained equations that has been performed up to now.

## 1. Introduction

Missing data is a major problem in medicine and other branches of science [1,2,3,4]. There are many competing methods to deal with missing data. Complete-case analysis is simple to implement, but has undesirable statistical properties. Maximum-likelihood estimation has good statistical properties, but requires implementation of whole algorithms, since there are usually no analytical expression in the presence of missing data. Even worse, these algorithms are rarely implemented in statistical software packages, so this implementation has to be done by the scientist who wants to analyze the data. Imputation methods, i.e., algorithms that generate values and impute them for the values missing in the data, are available in standard software packages and may have good statistical properties depending on their exact specification.

There are two kinds of imputation methods for data sets that have missing values in several variables, joint modeling and multiple imputation by chained equations [5]. In joint modeling one builds one multivariate model for all variables jointly. This method is computationally expensive, unless a multivariate normal distribution is suitable as joint model. Multiple imputation by chained equation [6], also called fully conditional specification or sequential regression, is an imputation method for which only marginal distributions of the variables given all other variables have to be specified. It works by imputing the variables one by one using a subroutine. This subroutine can be an arbitrary algorithm for imputing values in a data set in which only one variable has missing values. Multiple imputation by chained equations is available through the R-package mice [7].

There are many competing subroutines for multiple imputation by chained equation. Predictive mean matching [8], weighted predictive mean matching [9], simple sampling, classification and regression trees [10] and random forests [11] work for any kind of numerical data. For continuous variables unconditional mean imputation and (Bayesian) linear regression [12, p. 167, 13] are useful. For binary variables one can use logistic regression [14,15]. A survey is given in [6].

We consider three different missing data mechanisms: missing completely at random (MCAR), missing at random (MAR) and missing not at random (MNAR). These mechanisms assume that there is some unobserved full data of which several values are removed before the rest is observed. Under MCAR the choice whether a value is removed is made without regarding the data. Under MAR the choice whether a value is removed may depend on other observed values, however it may not depend on the values to be decided or on further unobserved values. Under MNAR the choice whether a value is removed may depend on the whole data and, in particular, on the value itself.

There are a lot of papers comparing different methods for treating missing data, e.g., [5,14,15,16,17,18,19,20,21,22,23] or the literature cited in [24]. In this article we take the point of view that the decision to treat the missing values using multiple imputation by chained equations has already been made and it only remains to choose the subroutine. Such comparisons have been made before in [25] for Cox regression with MCAR and MAR data and in [11] for linear regression and logistic regression with MAR data. Here we perform a big simulation study considering the estimation of means, the estimation of variances and covariances, linear regression, logistic regression and Cox regression for MCAR, MAR and MNAR data. Moreover, we apply the methods to data of patients suffering from an obstructive coronary artery disease (CAD). This is the first time that a really big simulation study concerning the choice of a subroutine for multiple imputation by chained equations is conducted and this is the first time that various multiple imputation algorithms are compared on data of patients suffering from obstructive CAD.

Multiple imputation by chained equations has certain limitations. On the theoretical side it is not satisfactory that parameters estimated based on imputed data do not converge to the true values as both the number of iterations and the sample size tend to infinity even under the MCAR assumption. Nevertheless, the estimated parameters get so close to the true parameters that there is not a practical problem. Moreover, under MNAR assumptions the difference may be so big that it is even a problem from a practical point of view. Finally the results of multiple imputation by chained equation depend on the choice of the subroutine, while this dependence is largely unexplored. The present paper will help understanding this dependence.

We consider 15 different simulation scenarios. Under each scenario we simulate 1,000 data sets and based on these data sets we calculate six statistics for each variable and each subroutine. With 15 simulation scenarios and six statistics per variable and subroutine this is a relative big simulation study. 1,000 simulated data sets are enough for a reasonable statistical analysis. As for every simulation study, our results are random to a certain degree. Moreover, a simulation study only shows that an algorithm performs good or poor without giving an explanation why.

We only include subroutines that are implemented in the R package mice. Since we will deal both with continuous and with categorical variables in this study, we consider only subroutines that can deal with both of them. Thus five subroutines qualified: predictive mean matching, weighted predictive mean matching, sample, classification or regression trees and random forests.

The rest of this paper is organized as follows. In Section 2 we explain the algorithms compared in this paper. Section 3 provides details on the real data. In Section 4 we report the setup and the results of our simulation study. In Section 5 we discuss the results and point out directions for future research.

## 2. Algorithms

Multiple imputation by chained equations is an algorithm for creating imputations in a data set which has missing values in several variables. It is an iterative algorithm. In the initialization for all missing values just a random value sampled uniformly from all observed values of the same variable is imputed (while it is remembered which values are real and which values are imputed). In the iteration step one variable is chosen and all its imputed values are replaced by new imputed values which are created using a subroutine. Such a subroutine is an algorithm which imputes values in a data set which has missing values in only one variable. Several possible choices for the subroutine will be given below. In order to apply the subroutine, for the chosen variable all values that have been implemented before are removed, while for all other variables the previously implemented values are treated as if they were real. Then the subroutine creates an imputation for the missing values of the variable at hand. All variables with missing values are treated one by one like this and, when all variables have been treated, one starts again with the first variable. This procedure is repeated several times – five times in this paper, in the R package mice it can be specified by the parameter maxit – and hence one completed data set is obtained. Since it is desirable to have multiple completed data sets [1,2,3,6], we have to run the whole algorithm multiple times – in this article 20 times, in mice to be specified by the parameter m.

A first possible subroutine is predictive mean matching (PMM). A regression model is fitted with the incomplete variable as target and all other variables as covariables based on the cases for which also the incomplete variable is observed using Bayesian regression. Then for each case in which the incomplete variable is missing, a value $\dot{y}$ is sampled from its posterior distribution and one looks for the five cases in which the incomplete variable is non-missing whose predicted value is closest to $\dot{y}$. Then one of the observed values of these five cases is chosen at random and inserted for the missing value. Notice that predictive mean matching has an extension to several variables with missing values, but this is not advised if there is only a small number of complete cases. PMM is the default subroutine of mice. Further information can be found in [8] or in [6, Section 3.4].

Weighted predictive mean matching, also called MIDAStouch, is a modified version of predictive mean matching. Instead of considering those cases whose predicted values have a small enough distance to $\dot{y}$ with equal probability, we now consider all cases, but with a probability that is the smaller the larger the distance between its predicted value and $\dot{y}$ is. An advantage over PMM is that the probability for an observed value to be chosen now decreases smoothly with the distance between its predicted value and $\dot{y}$ instead of dropping down to 0 at an arbitrarily chosen threshold. The price for this is an enormous increase in computation time. Further information can be found in [9].

The method called SAMPLE simply samples from all observed values of the incomplete variable without considering the information the other variables give. Notice that one gets the same results if you use its multivariable version directly as you get if you use it as a subroutine of multiple imputation by chained equations.

Classification or regression trees (CART) are trees used for the prediction of a target variable. At each node of the tree a covariable of the data set and a threshold is given and depending on whether this covariable has for the data item at hand a value which is less or greater than the threshold, one goes to the left child or to the right child of the node. One does this until one reaches a leaf. Then a good prediction of an unknown value of the target variable is possible by considering known values of the target variable of cases that end up in the same leaf. In order to use classification or regression trees in the context of imputation of missing data, one takes the variable which has missing values as target variable and all other variables as covariables. The tree is build using a training set which consists of all cases for which the incomplete variable is observed. For every node of the decision tree one selects the variable and the threshold yielding the best possible split (in the sense of maximal Gini impurity). After the tree is build, for each data item for which the incomplete variable is missing the leaf it ends up is determined and the imputed value is sampled randomly from all observed values belonging to that leaf. The CART method has been designed to be used as a subroutine of multiple imputation by chained equations and therefore it cannot deal directly with data that has missing values in more than one variable. The CART method can deal with non-linear effects, but it produces predictions that are locally constant. For further information on classification and regression trees in general see [26] or [27, Chapters 9–10] and for the use as imputation method see [10] or [6, Section 3.5].

Random forests (RF) consist of multiple classification or regression trees. In building the trees there are two differences to single trees: First as training data not the set of cases for which the incomplete variable is observed is taken, but a bootstrap sample of it is used. Second at each node a random subset of some variables is drawn and the optimal split is only determined among these variables. Now for each data item and each tree one has one leaf in which the data item ends up. The imputed value for a data item for which the incomplete variable is missing is now sampled from the union over all trees of all observed values of data items ending up in the same leaf. Similarly as CART, RF can deal with non-linear effects, but produces predictions that are locally constant. Further information on random forests in general can be found in [28] and [29] and for the use of random forests as imputation method we refer to [11] and [6, Section 3.5].

We excluded one subroutine that is suitable both for continuous and for categorical data, namely imputation at level 2 by predictive mean matching, from the comparison. Indeed, this method was designed to deal with multilevel data [6, Section 7.2 and Section 7.8], while the other methods in this comparison are not suitable for dealing with multilevel data, and hence any comparison would be questionable.

The experiments were carried out using R 4.2.0 with mice 3.14.0 and R 4.2.1 with mice 3.14.0.

## 3. The cardiologic data

In this section we apply the algorithms introduced in the previous section to real data.

We consider the problem of predicting mortality from seven risk factors (age, sex, systolic blood pressure, LDL, smoking behavior, diabetes, family history of premature heart diseases) after the diagnosis of an obstructive CAD. For this, we used data from the Essen coronary artery disease (ECAD) registry which contains the results of 33,978 coronary angiographies conducted between 01/01/2005 and 31/12/2019 at the Department for Cardiology and Vascular Medicine, University Hospital of Essen. In 10,627 coronary angiographies an obstructive coronary artery disease was discovered. These 10,627 coronary angiographies were conducted on 7,398 different patients and we included only the first positive coronary angiography for each patient. Finally one patient was excluded, since all variables we are interested in are missing, leaving 7,397 patients for the analysis.

We examined the follow-up duration, the information whether the patient died and the seven risk factors mentioned above. Of the 7,397 rows of the table, only 1,297 (18%) are complete. Statistics for the different columns of the data are shown in Table 1.

**Table 1:** Statistics of the different columns.

Column	count_NA	mean	mean_CC
follow-up duration	0 (0%)	3.368	3.367
death	0 (0%)	0.165	0.128
age	0 (0%)	65.437	65.790
sex	0 (0%)	0.749	0.748
systolic blood pressure	4250 (57%)	139.057	139.019
LDL	3094 (42%)	111.327	110.876
diabetes	4533 (61%)	0.274	0.266
Smoking	2775 (38%)	0.303	0.339
Family history	2768 (37%)	0.231	0.311

For each column of the table we report number and frequency of missing values (count_NA), the mean value of all data items for which the variable at hand was observed (mean) and the mean value of the 1,297 data items for which all values were observed (mean_CC).

We see that four variables are complete, while for the other variables up to 61% of the values are missing. The mean value for all patients for which the examined variable are known and the mean value for all patients for which all variables are known are close together except for the family history of premature heart diseases.

A natural question is, whether this data set follows an MCAR, an MAR or an MNAR mechanism. Since MNAR is known to be not testable, we only test the MCAR assumption assuming that MAR holds [4, Section 1.9]. In Table 2 we test for each pair of Quantity i and Quantity j whether Quantity i influences the missingness of Quantity j. In order to correct for multiple testing we applied the Bonferroni method, i.e., we multiplied all p-values by 45. We see many significant results implying that we do not have a MCAR mechanism.

**Table 2:** Test MCAR vs. MAR.

quantity	systolic blood pressure	LDL	diabetes	Smoking	Family history
follow-up duration	2.59e-05	1.00e + 00	9.21e-09	0.00e + 00	0.00e + 00
death	0.00e + 00	0.00e + 00	2.35e-10	7.07e-10	3.72e-12
age	6.58e-02	5.99e-06	1.00e + 00	8.61e-04	3.03e-03
sex	1.11e-02	1.00e + 00	1.00e + 00	2.60e-04	4.06e-04
systolic blood pressure		1.00e + 00	1.00e + 00	3.19e-01	6.76e-01
LDL	1.00e + 00		4.55e-01	1.00e + 00	1.00e + 00
diabetes	1.00e + 00	1.00e + 00		1.00e + 00	1.00e + 00
Smoking	6.70e-08	4.13e-01	9.48e-02		9.39e-06
Family history	9.67e-13	5.03e-04	1.82e-04	1.00e + 00	

For each pair of Quantity i (in the rows) and Quantity j (in the columns) we test whether Quantity i influences the missingness of Quantity j by testing Quantity i for those patients for which Quantity j is missing vs. those patients for which Quantity j is non-missing. We apply a t-test if Quantity i is approximately normal distributed (age, systolic blood pressure, LDL), a Wilcoxon test if Quantity i is seriously skewed (follow-up duration) and a chi-squared test if Quantity i is binary (death, sex, diabetes, smoking, family history).

We now compare the performance of the five subroutines mentioned in Section 2, when the analysis to be carried out is

- the calculation of the mean values of the variables,
- the calculation of the variances and covariances of the variables,
- the estimation of the coefficients in a logistic regression model or
- the estimation of the coefficients in a Cox regression model.

We do not consider linear regression here, since our data does not contain an appropriate target variable for that.

We analyze the data set described above, where the missing values are imputed using each of the five subroutines from Section 2 and producing M = 20 completed data sets (per subroutine). We report for each variable and each subroutine the mean value,

the inner standard deviation, the between standard deviation and the total standard deviation. In order to define these quantities precisely, let $m_{j,k}$ be the estimated value for the j -th variable and the k -th completed data set and let $s_{j,k}$ be the estimator for the standard deviation of the estimated value for the j -th variable based on the k -th completed data set. E.g., when the characteristic of interest is the expected value, then $m_{j,k}$ is the arithmetic mean over all patients, while $s_{j,k}$ is the empirical standard deviation divided by the square root of the number of patients. Then the mean value is defined as $m_{j}=\frac{1}{M}\cdot \sum_{k=1}^{M}m_{j,k}$, the inner standard deviation is defined as $si_{j}=\sqrt{\frac{1}{M}\cdot \sum_{k=1}^{M}s_{j,k}^{2}}$ and the between standarddeviation is $sb_{j}=\sqrt{\frac{1}{M-1}\cdot \sum_{k=1}^{M}{\left(m_{j,k}-m_{j}\right)}^{2}}$. The total standard deviation is calculated using Rubin’s formula (see, e.g., (5.20) in [1]).


$$st_{j}=\sqrt{si_{j}^{2}+\left(1+\frac{1}{M}\right)\cdot sb_{j}^{2}}.$$

For categorical variables we additionally evaluated the number of imputations that were nonsense, meaning that a value which does not make sense for this variable was imputed.

The results for the estimation of the expected values of systolic blood pressure and of diabetes are reported in Table 3. Since the true values are unknown, all that can be said for systolic blood pressure is that the values are plausible. The same holds for LDL, smoking behavior and family history and therefore the results for these variables were moved to the supplementary material. A special situation holds, however, for diabetes. The test for diabetes was not conducted if it was obvious that the patient did not suffer from diabetes. So the presence of diabetes reduced the probability that the diabetes variable is missing. This is missing not at random (MNAR). It is better to impute the value 0 (no diabetes) for missing values than to use any of the five subroutines tested here. This way, we get a mean value of 10.60% which is less than any of the five values in Table 3 by far.

**Table 3:** Estimates for the expected values of the real data example.

Method	mean	inner	between	total	
Systolic blood pressure					
pmm	138.7	0.261	0.286	0.392	
midastouch	138.6	0.261	0.284	0.391	
sample	139.0	0.260	0.185	0.322	
cart	138.3	0.265	0.264	0.379	
rf	138.9	0.260	0.170	0.313	
Method	mean	inner	between	total	nonsense
Diabetes					
pmm	0.2787	0.00521	0.00509	0.00738	0
midastouch	0.2785	0.00521	0.00682	0.00872	0
sample	0.2738	0.00518	0.00523	0.00745	0
cart	0.2847	0.00525	0.00518	0.00747	0
rf	0.2657	0.00514	0.00589	0.00792	0

None of the five subroutines did ever impute a value that was nonsense. So it is justified that these five subroutines are claimed in the R help files to work for categorical data.

In Table 4 we present the computation times of the different algorithms for the generation of all 20 imputations for all variables in the example just considered. The experiment was carried out on an Intel Core i7-11700 processor with 8 cores and 2.50 GHz. We see that SAMPLE is the fastest subroutine and PMM is neglectable slower. The computation times for CART and RF are already more than 10 times as high, but still they are less than 4% of the computation time for MIDAStouch. Considering that a RF consist of multiple trees, it is quite surprising that RF is faster than CART.

**Table 4:** Computation time of the mice algorithm with the different subroutines in seconds.

Method	Time
pmm	4.889
midastouch	2244.210
sample	3.382
cart	79.624
rf	55.093

The results for the estimation of variances and for fitting logistic regression model and Cox regression models are deferred to the supplementary material. They brought essentially the same conclusion as the results for the estimation of expected values.

## 4. Simulations

### 4.1. Expected values.

Next we want to evaluate the subroutines on simulated data. While the model for the simulated data is designed to share some features of the real data, we do not aim at getting as close to the real data generation process as possible, and, in particular, we do not estimate values in order to fit the model. We define


$$\left(\begin{array}{c} Y_{1}\\ Y_{2}\\ Y_{3}\\ Y_{4}\\ Y_{5}\\ Y_{6}\\ Y_{7}\\ Y_{8}\\ Y_{9} \end{array}\right)=\left(\begin{array}{c} \exp\left\{X_{1}\right\}\\ 1_{\left\{{\text{X}}_{2}>1\right\}}\\ 65+10\cdot X_{3}\\ 1_{\left\{{\text{X}}_{4}>-1\right\}}\\ 140+20\cdot {\text{X}}_{5}\\ 110+20\cdot X_{6}\\ 1_{\left\{{\text{X}}_{7}>1\right\}}\\ 0.5\cdot 1_{\left\{{\text{X}}_{8}>0\right\}}+0.5\cdot 1_{\left\{{\text{X}}_{8}>1\right\}}\\ 1_{\left\{{\text{X}}_{9}>0.5\right\}} \end{array}\right)\;,$$

where $\left(X_{1},\ldots ,\;X_{9}\right)$ is multivariate normal distributed with mean zero and covariance matrix


$$\left(\begin{array}{ccccccccc} 1 & 0.2 & 0.2 & -0.3 & -0.2 & 0 & 0 & -0.2 & 0.2\\ 0.2 & 1 & 0.9 & 0.1 & 0.4 & 0.2 & -0.2 & 0 & 0\\ 0.2 & 0.9 & 1 & 0.1 & 0.4 & 0.2 & -0.2 & 0.1 & -0.1\\ -0.3 & 0.1 & 0.1 & 1 & -0.2 & 0.1 & 0.1 & 0.4 & 0.5\\ -0.2 & 0.4 & 0.4 & -0.2 & 1 & -0.4 & 0.3 & 0.1 & -0.1\\ 0 & 0.2 & 0.2 & 0.1 & -0.4 & 1 & 0 & 0.3 & -0.2\\ 0 & -0.2 & -0.2 & 0.1 & 0.3 & 0 & 1 & 0.5 & 0.2\\ -0.2 & 0 & 0.1 & 0.4 & 0.1 & 0.3 & 0.5 & 1 & -0.3\\ 0.2 & 0 & -0.1 & 0.5 & -0.1 & -0.2 & 0.2 & -0.3 & 1 \end{array}-\right)$$

We consider three different missing data mechanisms: missing completely at random (MCAR), missing at random (MAR) and missing not at random (MNAR). These mechanisms assume that there is some unobserved full data of which several values are removed before the rest is observed – and that is the way we proceed in the simulations. Under MCAR the choice whether a value is removed is made without regarding the data. We removed $Y_{5}$ and $Y_{7}$ with probability 0.6 and $Y_{6},Y_{8}$ and $Y_{9}$ with probability 0.3 independent of each other and independent of the random variables $Y_{1},Y_{2},\ldots ,\;Y_{9}$. Under MAR the choice whether a value is removed may depend on other observed values, however it may not depend on the values to be decided or on further unobserved values. We removed $Y_{5}$ if $Y_{3}$ was greater than its 0.4 -quantile, $Y_{6}$ and $Y_{8}$ with probability $\frac{0.3}{\;\;\;\Phi \;\;\;\left(1\right)}$ if $Y_{4}=1$, $Y_{7}$ with probability 0.8 if $Y_{3}>65$ and with probability 0.4 if $Y_{3}\leq 65$ and $Y_{9}$ with probability 0.4 if $Y_{4}=0$ and with probability $0.4-\frac{0.1}{\;\;\;\Phi \;\;\;\left(1\right)}$ if $Y_{4}=1$, where all events in this sentence for which only a (conditional) probability is specified should be (conditionally) independent of $Y_{1},\ldots ,\;Y_{9}$ and independent of each other and where  Φ  denotes the cumulative distribution function of the standard normal distribution. Under MNAR the choice whether a value is removed may depend on the whole data and, in particular, on the value itself. We removed $Y_{5}$ if it is greater than the 0.4 -quantile of its distribution, $Y_{6}$ with probability 0.4 if $Y_{6}>110$ and with probability 0.2 if $Y_{6}\leq 110$, $Y_{7}$ with probability 0.6/ Φ (1) if $Y_{7}=0$, $Y_{8}$ with probability 0.6 if $Y_{8}>0$, and $Y_{9}$ with probability 0.4 if $Y_{9}=0$ and with probability 0.4−0.1/ Φ (−0.5) if $Y_{9}=1$. Notice that these probabilities were arranged in such a way that for each variable the probabilities that it is missing under MCAR, MAR or MNAR are the same.

Due to the enormous computation time of MIDAStouch we did not apply this subroutine to the simulated data, but we focused on the other four methods. It is an open problem to write an algorithm with same statistical properties as MIDAStouch and an acceptable computation time.

We simulate a dataset of 10,000 patients and we repeat the experiments n=1,000 times. For each variable and each subroutine, we report the mean, the standard deviation using Rubin’s formula, the absolute bias, the simulated standard deviation, the square root of the mean squared error and the coverage probability of the confidence interval. In order to make this precise let $m_{j,k,l}$ and $s_{j,k,l}$ be the fitted value and the estimator of its standard deviation for the j -th variable in the k -th completed data set of the l -th repetition and let $\mu_{j}$ denote the true value. Then


$$m_{j}=\frac{1}{n}\sum_{l=1}^{n}m_{j,l},\;m_{j,l}=\frac{1}{M}\sum_{k=1}^{M}m_{j,k,l},$$

$$st_{Rubin,\;j\;}=\sqrt{\frac{1}{n}\cdot \sum_{l=1}^{n}st_{Rubin,j,l}^{2},}$$

$$\;st_{Rubin,\;j,l\;}=\sqrt{\frac{1}{M}\sum_{k=1}^{M}s_{j,k,l}^{2}+\left(1+\frac{1}{M}\right)\frac{1}{M-1}\sum_{k=1}^{M}{\left(m_{j,k,l}-m_{j,l}\right)}^{2}}\;$$

$$b_{j}=\frac{1}{n}\sum_{l=1}^{n}\left|m_{j,l}-\mu_{j}\right|,\;$$

$$st_{simulated,\;j}=\sqrt{\frac{1}{n-1}\sum_{l=1}^{n}{\left(m_{j,l}-m_{j}\right)}^{2}},$$

$$MSE_{j}=\frac{1}{n}\sum_{l=1}^{n}{\left(m_{j,l}-\mu_{j}\right)}^{2},$$

$$Cover_{j}=\frac{1}{n}\sum_{l=1}^{n}1_{\left(m_{j,l}+z_{\frac{\alpha }{2}}\cdot st_{Rubin,j,l},m_{j,l}+z_{1-\frac{\alpha }{2}}\cdot st_{Rubin,j,l}\right)}\left(\mu_{j}\right),\;\alpha =0.05,\;$$

where $z_{\alpha }$ denotes the α -quantile of the standard normal distribution.

The true means $\mu_{j}$ (needed to determine the coverage probability of the confidence intervals) were calculated analytically.

In order to save space only the results for Variable 5 are included in Table 5, while the results for the other variables are deferred to the supplementary material.

**Table 5:** Simulation results for the expected values.

Method	mean	sd_Rubin	Absolute_bias	Simulated_sd	sqrt_MSE	Coverage
pmm	140.0	0.274	0.216	0.271	0.271	0.946
sample	140.0	0.255	0.252	0.318	0.318	0.875
cart	139.9	0.249	0.225	0.272	0.284	0.909
rf	139.7	0.364	0.349	0.301	0.425	0.898
Method	mean	sd_Rubin	Absolute_bias	Simulated_sd	sqrt_MSE	Coverage
pmm	140.9	1.445	1.32	1.444	1.73	0.828
sample	132.3	0.241	7.73	0.307	7.73	0.000
cart	133.1	0.562	6.95	0.564	6.97	0.000
rf	132.0	0.538	8.03	0.345	8.04	0.000
Method	mean	sd_Rubin	Absolute_bias	Simulated_sd	sqrt_MSE	Coverage
pmm	123.6	0.162	16.4	0.169	16.4	0
sample	120.7	0.142	19.3	0.176	19.3	0
cart	123.3	0.169	16.7	0.178	16.7	0
rf	122.0	0.263	18.0	0.171	18.1	0

These are the results for Variable 5. In the first table the results for MCAR data are presented, in the second table the results under MAR assumptions are shown and, in order to obtain the third table, we assume MNAR.

Under MCAR we see that the mean value is close to the true value for all algorithms and all variables, likewise the standard deviation from Rubin’s formula is close to the simulated standard deviation and the coverage probability of the confidence intervals is close to the nominal level. There is one exception, namely RF yields poor results for Variable 7, while it is not obvious what the problem is with this combination. Moreover, in general the results for PMM are a bit better than the results for the other subroutines.

Under MAR the superiority of PMM is becoming quite pronounced, with CART usually taking the second place. This is a bit surprising, since RF is more sophisticated than CART. Under MNAR all methods behave poorly. Usually the mean values of PMM and CART are a bit closer to the true values than the mean values of SAMPLE or RF, but the difference between the subroutines is small compared to the distance any of these mean values has to the true value.

## 4.2. Variances and covariances

After having analyzed the mean values in Section 4.1, the logical next step is the analysis of variances and covariances. We use the same simulation model as in Section 4.1. Due to the large amount of results, only variances will be included in the main article and the pdf supplementary material and the covariances will only be given in the supplementary txt-files.

Notice that unlike in Section 4.1, the true values are not readily available. Hence we generated a sample of 1,000,000 patients without missing data and took the empirical variances and covariances of that sample as “real” variances and covariances.

The results on Variable 5 are given in Table 6, the other results are deferred to the supplementary material. For MCAR data the situation for variances is similar to the situation for expected values. The best results are produced by PMM and the only really poor results are those by RF for Variable 7. In general, the coverage probabilities for variances are a bit lower than those for mean values.

**Table 6:** Simulation results for the variances.

Method	mean	sd_Rubin	Absolute_bias	Simulated_sd	sqrt_MSE	Coverage
pmm	399.3	8.06	6.92	8.71	8.75	0.917
sample	399.4	7.21	7.31	9.23	9.26	0.872
cart	397.2	7.40	7.42	8.78	9.28	0.867
rf	385.6	7.95	14.95	8.64	16.97	0.545
Method	mean	sd_Rubin	Absolute_bias	Simulated_sd	sqrt_MSE	Coverage
pmm	435.0	37.86	38.0	39.98	53.0	0.88
sample	356.1	6.43	44.1	7.84	44.8	0.00
cart	345.5	14.16	54.7	13.76	56.4	0.06
rf	340.5	8.50	59.7	7.91	60.2	0.00
Method	mean	sd_Rubin	Absolute_bias	Simulated_sd	sqrt_MSE	Coverage
pmm	95.56	2.45	305	2.44	305	0
sample	124.66	2.82	276	3.46	276	0
cart	97.36	2.65	303	2.67	303	0
rf	109.78	3.78	290	2.94	290	0

The details are the same as for Table 5.

Similar things can be said about MAR data. The results for PMM and CART are better than the results for SAMPLE and RF as we already had it for the mean values. The results of Variables 5 are quite poor. The coverage probabilities for variances are a bit lower than those for mean values.

Under MNAR we obtain good results for Variable 6 and poor results for the other variables. An explanation, what is so special about Variable 6, is not obvious.

## 4.3. Linear regression model

We fitted a linear regression model whose covariables were the third to ninth variable $Y_{3},\ldots ,\;Y_{9}$ of Section 4.1 and whose target variable was


$$Z=Y_{3}+20\cdot Y_{4}+0.2\cdot Y_{5}-0.2\cdot Y_{6}+5\cdot Y_{7}-10\cdot Y_{8}+Y_{9}+100+\epsilon ,$$

where $\epsilon \sim 𝒩\left(0,10^{2}\right).$ The variables $Y_{3},\ldots ,\;Y_{9}$ were missing as in Section 4.1 and Z was never missing.

The results for Variable 5 are presented in Table 7, the other results are deferred to the supplementary material. For MCAR data, PMM produces good results, while the estimates of CART are slightly biased yielding a low coverage probability. For SAMPLE the situation is even worse resulting in a coverage probability of 0. This may be due to the fact that SAMPLE does not make attempts to reconstruct dependencies between different variables. Frequently, Rubin’s formula overestimates the standard deviation and this results in a too high coverage probability for the coefficients of $Y_{3},Y_{4}$ and $Y_{5}$ for RF.

**Table 7:** Simulation results for linear regression models.

Method	mean	sd_Rubin	Absolute_bias	Simulated_sd	sqrt_MSE	Coverage
pmm	0.20254	0.01206	0.00992	0.01219	0.0124	0.933
sample	0.08201	0.00789	0.11799	0.00353	0.1180	0.000
cart	0.20245	0.01106	0.01113	0.01389	0.0141	0.847
rf	0.17460	0.02578	0.02541	0.00816	0.0267	0.992
Method	mean	sd_Rubin	Absolute_bias	Simulated_sd	sqrt_MSE	Coverage
pmm	0.20262	0.01253	0.0103	0.01267	0.0129	0.939
sample	0.09004	0.00826	0.1100	0.00396	0.1100	0.000
cart	0.15502	0.02130	0.0451	0.01708	0.0481	0.407
rf	0.14405	0.02523	0.0560	0.00863	0.0566	0.291
Method	mean	sd_Rubin	Absolute_bias	Simulated_sd	sqrt_MSE	Coverage
pmm	0.16232	0.0197	0.0380	0.01822	0.0419	0.518
sample	0.07785	0.0137	0.1221	0.00617	0.1223	0.000
cart	0.15702	0.0201	0.0433	0.02112	0.0479	0.440
rf	0.16786	0.0288	0.0322	0.01230	0.0344	0.922

The details are the same as for Table 5.

All this remains true under MAR assumptions. Under MNAR assumptions all algorithms behave poorly.

## 4.4. Logistic regression model

Here we considered a logistic regression model with covariables $Y_{3},\ldots ,Y_{9}$ as in Section 4.1 and with a target variable I fulfilling


$$\mathbb{P}\left(I=1\right)=\frac{\exp\left(Z\right)}{\exp\left(Z\right)+1},$$

where


$$Z=(Y_{3}+20\cdot Y_{4}+0.2\cdot Y_{5}-0.2\cdot Y_{6}+5\cdot Y_{7}-10\cdot Y_{8}+Y_{9}-100)/20.$$

The variables $Y_{3},\ldots ,\;Y_{9}$ were missing as in Section 4.1 and I was never missing.

The results for Variable 5 are presented in Table 8, the remaining results are deferred to the supplementary material. Both under MCAR and under MAR assumptions, we get good results of PMM, poor results of SAMPLE and the other two methods being in between. For MNAR data, there are some good results among a lot of poor ones, but these good results are so irregularly scattered that it is probably pure coincidence. We remark that the coverage probabilities we obtain here are higher than the coverage probabilities we obtained for estimating the expected values or the variances or for linear regression models under MNAR assumptions, but still unacceptable low.

**Table 8:** Simulation results for logistic regression models.

Method	mean	sd_Rubin	Absolute_bias	Simulated_sd	sqrt_MSE	Coverage
pmm	0.010163	0.00283	0.00222	0.002834	0.00284	0.934
sample	0.004009	0.00146	0.00599	0.000698	0.00603	0.000
cart	0.007038	0.00225	0.00308	0.001841	0.00349	0.777
rf	0.012899	0.00268	0.00296	0.001793	0.00341	0.906
Method	mean	sd_Rubin	Absolute_bias	Simulated_sd	sqrt_MSE	Coverage
pmm	0.010136	0.00307	0.00250	0.003125	0.00313	0.937
sample	0.003805	0.00156	0.00620	0.000689	0.00623	0.000
cart	0.006281	0.00376	0.00402	0.002614	0.00455	0.824
rf	0.009822	0.00322	0.00152	0.001886	0.00189	0.994
Method	mean	sd_Rubin	Absolute_bias	Simulated_sd	sqrt_MSE	Coverage
pmm	0.006881	0.00393	0.00390	0.00359	0.00476	0.887
sample	0.002893	0.00262	0.00711	0.00115	0.00720	0.050
cart	0.006089	0.00383	0.00431	0.00319	0.00505	0.848
rf	0.012530	0.00427	0.00335	0.00330	0.00416	0.971

The details are the same as for Table 5.

## 4.5. Cox regression

We simulated a Cox regression model with covariables $Y_{3},\ldots ,\;Y_{9}$ as in Section 4.1, a survival time that was exponentially distributed with rate 1/(20·exp{Z}), where


$$Z=(Y_{3}+20\cdot Y_{4}+0.2\cdot Y_{5}-0.2\cdot Y_{6}+5\cdot Y_{7}-10\cdot Y_{8}+Y_{9}-100)/20,$$

and a competing risk whose time point is exponentially distributed with rate 1/5 independent of the covariables.

We present the results on Variable 5 in Table 9 and the remaining results in the supplementary material. Once again, both under MCAR and under MAR the best method is PMM. Under MNAR we observe the same as in logistic regression: Among a lot of poor results there are some irregularly scattered good ones.

**Table 9:** Simulation results for Cox regression models.

Method	mean	sd_Rubin	Absolute_bias	Simulated_sd	sqrt_MSE	Coverage
pmm	0.009321	0.00207	0.001701	0.002013	0.00212	0.936
sample	0.004028	0.00113	0.005972	0.000517	0.00599	0.000
cart	0.006916	0.00170	0.003108	0.001431	0.00340	0.550
rf	0.010353	0.00176	0.000946	0.001121	0.00117	0.997
Method	mean	sd_Rubin	Absolute_bias	Simulated_sd	sqrt_MSE	Coverage
pmm	0.011451	0.00198	0.00199	0.001998	0.00247	0.884
sample	0.005687	0.00115	0.00431	0.000648	0.00436	0.001
cart	0.007781	0.00232	0.00242	0.001740	0.00282	0.886
rf	0.009674	0.00198	0.00101	0.001220	0.00126	0.996
Method	mean	sd_Rubin	Absolute_bias	Simulated_sd	sqrt_MSE	Coverage
pmm	0.008584	0.00250	0.00213	0.002294	0.00269	0.918
sample	0.005028	0.00187	0.00497	0.000974	0.00507	0.096
cart	0.007471	0.00243	0.00275	0.002085	0.00328	0.855
rf	0.011089	0.00247	0.00172	0.001826	0.00213	0.979

The details are the same as for Table 5.

## 5. Discussion

We investigated multiple imputation by chained equations, a method for treating missing data. We compared five different subroutines, but one of them, MIDAStouch, had to be excluded from a part of our analysis due to its long computation time. Among the remaining four methods there is one that clearly performed best, namely predictive mean matching (PMM).

This generalizes the finding of [25] that at least under simple, “linear” interactions between the different components of the observed random vector, PMM is superior to tree-based methods. However, it was noticed both in [11] and in [25] that, in models with more complex, “non-linear” interactions between the different components of the observed random vector, CART and RF may be superior to PMM.

Non-linear interactions can arise in different ways, e.g., a variable can depend in a non-linear way on another variable or one variable can depend on the product of two variables. In the data set from our cardiologic data example, we have, however, not much problems with that. In a quadratic regression model with systolic blood pressure as target variable and age as covariable we got a p-value of 0.056 for complete case analysis, 0.113 for PMM, 0.270 for SAMPLE, 0.143 for CART and 0.133 for RF for the hypothesis that the true quadratic coefficient is zero. Hence we have no reason to assume a non-linear relation. In a logistic regression model with death as target variable and age, diabetes and the product of age and diabetes as covariables we got a p-value of 0.063 for complete case analysis, 0.390 for PMM, 0.826 for SAMPLE, 0.489 for CART and 0.126 for RF for the hypotheses that the true product coefficient is zero. Hence we have no reason to assume a non-linear relation here either. The results are similar for most other variables.

The results for MNAR are poor throughout – as could be expected due to similar findings in the literature. In such a case specific model assumptions have to be made [1, Chapter 15], e.g., the probit selection model or the normal pattern-mixture model.

In several occasions, Rubin’s formula severely overestimated the standard deviation even under MCAR assumptions for all subroutines except PMM – in particular in linear regression models, but also in logistic and Cox regression models.

We had to restrict ourselves to a relatively small study in order to keep the paper to an acceptable length. Besides the type of the target variable and the missing data mechanism also the amount of missingness, the number of variables and the number of data items may have an impact on the performance of a multiple imputation algorithm. It would have been desirable to examine this, but if we had tried out only three values for each of these influence factors, we would have ended up with a paper containing 27 times as many tables.

A further restriction is that we considered only one model per target and missing data mechanism. As mentioned five paragraphs ago, the performance of a multiple imputation algorithm may depend on the exact model. So it would have been desirable to try out several different models per target and missing data algorithm.

We considered only correctly specified models in our simulation. It would be interesting to consider misspecified models as well. Model misspecifications can arise easily, e.g., by fitting a (generalized) linear model when the true interactions are non-linear or by choosing an incorrect link function.

We restricted ourselves to those subroutines that can handle both continuous and binary variables. There are 20 subroutines that can handle continuous data and 12 subroutines that can handle binary data implemented in the mice package. So while it would be desirable to include every combination of a subroutine for continuous data and a subroutine for binary data in the comparison, there are 240 such combinations.

Considering that there is a vast literature on comparisons of missing data treatment techniques, but every paper can only cover a relatively small study as explained in the last four paragraphs, some meta-analysis seems to be desirable.

The simulation results tell us *that* PMM is superior to the other methods, but they do not tell us *why*. Hence some theoretical analysis seems to be desirable.

## Supporting information

MI_comparison_supp_rev Additional tables as pdf. All tables referred to in this article – whether they were presented or not – can be found in this pdf file.
(PDF)

S2_Tables Additional tables as txt. The same content as MI_comparison_supp_rev in a form that it is readable by computers. The specification of the txt-files can be found in the last chapter of MI_comparison_supp_rev.
(ZIP)

MI_001_elementary.R R file for the elementary computations presented in Section 3. Some modifications have to be made before executing this file, since the data used in it is not publically available.
(R)

MI_002_ECAD_Mean.R R file for the mean values of the real data presented in Section 3. Some modifications have to be made before executing this file, since the data used in it is not publically available.
(R)

MI_003_generator.R R file that generates the random vectors used by the other R files. (R)

MI_004_Sim_Mean_A.R R file for the simulation of the mean values presented in Section 4.1. This file can be executed directly.
(R)

MI_005_Sim_Mean_B.R R file for the simulation of the mean values presented in Section 4.1. This file can be executed directly.
(R)

MI_006_ECAD_Var.R R file for the (co)variances of the real data presented in Section 3. Some modifications have to be made before executing this file, since the data used in it is not publically available.
(R)

MI_007_Sim_Var_A.R R file for the simulation of the (co)variances presented in Section 4.2. This file can be executed directly.
(R)

MI_008_Sim_Var_B.R R file for the simulation of the (co)variances presented in Section 4.2. This file can be executed directly.
(R)

MI_009_Sim_LinReg_A.R R file for the simulation of the linear regression presented in Section 4.3. This file can be executed directly.
(R)

MI_010_Sim_LinReg_B.R R file for the simulation of the linear regression presented in Section 4.3. This file can be executed directly.
(R)

MI_011_ECAD_LogReg.R R file for the logistic regression of the real data presented in Section 3. Some modifications have to be made before executing this file, since the data used in it is not publically available.
(R)

MI_012_Sim_LogReg_A.R R file for the simulation of the logistic regression presented in Section 4.4. This file can be executed directly.
(R)

MI_013_Sim_LogReg_B.R R file for the simulation of the logistic regression presented in Section 4.4. This file can be executed directly.
(R)

MI_014_ECAD_CoxReg.R R file for the Cox regression of the real data presented in Section 3. Some modifications have to be made before executing this file, since the data used in it is not publically available.
(R)

MI_015_Sim_CoxReg_A.R R file for the simulation of the Cox regression presented in Section 4.5. This file can be executed directly.
(R)

MI_016_Sim_CoxReg_B.R R file for the simulation of the Cox regression presented in Section 4.5. This file can be executed directly.
(R)
