# Supplementary material for: A comparison of various imputation algorithms for missing data
Source: PLoS One. 2025 May 12;20(5):e0319784. doi: 10.1371/journal.pone.0319784 (PMC12068701; doi:10.1371/journal.pone.0319784)
Supplement: MI_comparison_supp_rev — All tables referred to in this article – whether they were presented or not – can be found in this pdf file. (PDF) [file pone.0319784.s001.pdf]

Supplementary material to
“A comparison of various imputation algorithms for missing data”

Jürgen Kampf (1), Iryna Dykun (1), Tienush Rassaf (1), Amir Abbas Mahabadi (1)
(1) Department of Cardiology and Vascular Medicine, University Hospital of Essen, Essen, Germany

We provide all tables that were either included in the article or excluded from the article in order to keep it to an acceptable size, both here in the pdf-file and in txt-files. A specification for the txt-files can be found in the last chapter of this pdf-file.

1. Expected values

1.1. Elementary statistics

There are 7397 rows, of which 1297 are complete.

Statistics of the different columns

Column	count_NA	mean	mean_CC
followupdur	0 (0 %)	3.368	3.367
death	0 (0 %)	0.165	0.128
age	0 (0 %)	65.437	65.790
sex	0 (0 %)	0.749	0.748
SysRR	4250 (57 %)	139.057	139.019
LDL	3094 (42 %)	111.327	110.876
diab	4533 (61 %)	0.274	0.266
Smoking	2775 (38 %)	0.303	0.339
Family	2768 (37 %)	0.231	0.311

Covariance matrix (for every covariance all items for which the two involved columns were non-missing are used).

age	sex	SysRR	LDL	diab	Smoking	Family
1.36e+02	-8.17e-01	3.70e+01	-4.72e+01	5.63e-01	-1.47e+00	-7.43e-01
-8.17e-01	1.88e-01	-2.69e-01	-1.89e+00	-5.60e-03	1.96e-02	-1.67e-03
3.70e+01	-2.69e-01	5.01e+02	5.91e+01	5.98e-01	-7.08e-01	8.10e-02
-4.72e+01	-1.89e+00	5.91e+01	1.71e+03	-3.06e-01	-5.30e-02	4.53e-01
5.63e-01	-5.60e-03	5.98e-01	-3.06e-01	1.99e-01	-9.67e-03	-9.82e-03
-1.47e+00	1.96e-02	-7.08e-01	-5.30e-02	-9.67e-03	1.59e-01	3.14e-02
-7.43e-01	-1.67e-03	8.10e-02	4.53e-01	-9.82e-03	3.14e-02	1.78e-01

Covariance matrix of complete cases

age	sex	SysRR	LDL	diab	Smoking	Family
1.44e+02	-7.47e-01	2.93e+01	-7.43e+01	5.07e-01	-1.82e+00	-7.60e-01
-7.47e-01	1.89e-01	-2.50e-01	-1.41e+00	-1.56e-03	2.45e-02	-1.85e-03
2.93e+01	-2.50e-01	4.61e+02	1.47e+01	4.77e-01	-6.62e-01	8.22e-02
-7.43e+01	-1.41e+00	1.47e+01	1.58e+03	-5.83e-01	6.17e-01	7.04e-01
5.07e-01	-1.56e-03	4.77e-01	-5.83e-01	1.95e-01	-1.61e-02	-8.64e-03
-1.82e+00	2.45e-02	-6.62e-01	6.17e-01	-1.61e-02	1.62e-01	2.50e-02
-7.60e-01	-1.85e-03	8.22e-02	7.04e-01	-8.64e-03	2.50e-02	2.14e-01

Next we test the MCAR assumption assuming that MAR holds. For each pair of Quantity i (in the rows) and Quantity j (in the columns) we test whether Quantity i influences the missingness of Quantity j by testing Quantity i for those patients for which Quantity j is missing vs. those patients for which Quantity j is non-missing. We apply a t-test if Quantity i is approximately normal distributed (age, SysRR, LDL), a Wilcoxon test if Quantity i is seriously skewed (followupdur) and a chi-squared test if Quantity i is

binary (death, sex, diab, smoking, family). In order to correct for multiple testing we applied the Bonferroni method, i.e. we multiplied all p-values by 45.

quantity	SysRR	LDL	diab	Smoking	Family
followupdur	2.59e-05	1.00e+00	9.21e-09	0.00e+00	0.00e+00
death	0.00e+00	0.00e+00	2.35e-10	7.07e-10	3.72e-12
age	6.58e-02	5.99e-06	1.00e+00	8.61e-04	3.03e-03
sex	1.11e-02	1.00e+00	1.00e+00	2.60e-04	4.06e-04
SysRR		1.00e+00	1.00e+00	3.19e-01	6.76e-01
LDL	1.00e+00		4.55e-01	1.00e+00	1.00e+00
diab	1.00e+00	1.00e+00		1.00e+00	1.00e+00
Smoking	6.70e-08	4.13e-01	9.48e-02		9.39e-06
Family	9.67e-13	5.03e-04	1.82e-04	1.00e+00	

1.2. Analysis of mean values - real data

For each variable and each subroutine we report

- the mean of the predicted values,
- the standard deviation within the imputations (as given by the common variance estimator),
- the standard deviation between the imputations (the standard deviation of the predicted values from the different imputations),
- the total standard deviation (obtained from Rubin's formula) and
- for categorical variables: the number of the imputations that were nonsense (meaning that a number which does not make sense for the present variable was imputed).

Systolic blood pressure

Method	mean	inner	between	total
pmm	138.7	0.261	0.286	0.392
midastouch	138.6	0.261	0.284	0.391
sample	139.0	0.260	0.185	0.322
cart	138.3	0.265	0.264	0.379
rf	138.9	0.260	0.170	0.313

LDL

Method	mean	inner	between	total
pmm	111.2	0.480	0.440	0.659
midastouch	111.3	0.483	0.365	0.611
sample	111.3	0.479	0.382	0.618
cart	111.2	0.488	0.306	0.581
rf	111.0	0.479	0.304	0.571

Diabetes

Method	mean	inner	between	total	nonsense
pmm	0.2787	0.00521	0.00509	0.00738	0
midastouch	0.2785	0.00521	0.00682	0.00872	0
sample	0.2738	0.00518	0.00523	0.00745	0

Method	mean	inner	between	total	nonsense
cart	0.2847	0.00525	0.00518	0.00747	0
rf	0.2657	0.00514	0.00589	0.00792	0

Smoking behavior

Method	mean	inner	between	total	nonsense
pmm	0.3106	0.00468	0.00207	0.00513	0
midastouch	0.3100	0.00467	0.00261	0.00538	0
sample	0.3028	0.00464	0.00246	0.00528	0
cart	0.3133	0.00467	0.00313	0.00567	0
rf	0.3033	0.00464	0.00418	0.00631	0

Family history of premature CAD

Method	mean	inner	between	total	nonsense
pmm	0.2379	0.00495	0.00585	0.00778	0
midastouch	0.2388	0.00496	0.00275	0.00570	0
sample	0.2302	0.00489	0.00423	0.00654	0
cart	0.2432	0.00499	0.00369	0.00626	0
rf	0.2271	0.00487	0.00331	0.00593	0

Time

Method	Time
pmm	4.889
midastouch	2244.210
sample	3.382
cart	79.624
rf	55.093

1.3. Analysis of mean values - simulated MCAR data

For each variable and each subroutine we report

- the mean of all predicted values,
- the standard deviation according to Rubin’s formula,
- the absolute bias,
- the simulated standard deviation (i.e. the empirical standard deviation of the predicted values from the different repetitions),
- the square root of the mean squared error and
- the coverage probability of a confidence interval obtained from a normal approximation, where the standard deviation was calculated using Rubin’s formula.

Variable 5 mean = 140

Method	mean	sd_Rubin	Absolute_bias	Simulated_sd	sqrt_MSE	Coverage
pmm	140.0	0.274	0.216	0.271	0.271	0.946
sample	140.0	0.255	0.252	0.318	0.318	0.875
cart	139.9	0.249	0.225	0.272	0.284	0.909
rf	139.7	0.364	0.349	0.301	0.425	0.898

Variable 6 mean = 110

Method	mean	sd_Rubin	Absolute_bias	Simulated_sd	sqrt_MSE	Coverage
pmm	110	0.233	0.188	0.236	0.235	0.950
sample	110	0.230	0.193	0.243	0.243	0.931
cart	110	0.228	0.191	0.238	0.238	0.940
rf	110	0.241	0.197	0.242	0.246	0.946

Variable 7 mean = 0.1587

Method	mean	sd_Rubin	Absolute_bias	Simulated_sd	sqrt_MSE	Coverage
pmm	0.1591	0.00586	0.00450	0.00567	0.00568	0.951
sample	0.1588	0.00466	0.00462	0.00588	0.00588	0.871
cart	0.1591	0.00497	0.00449	0.00565	0.00566	0.906
rf	0.1433	0.00526	0.01535	0.00573	0.01635	0.197

Variable 8 mean = 0.3293

Method	mean	sd_Rubin	Absolute_bias	Simulated_sd	sqrt_MSE	Coverage
pmm	0.3294	0.00431	0.00347	0.00432	0.00432	0.957
sample	0.3294	0.00423	0.00354	0.00442	0.00442	0.946
cart	0.3295	0.00420	0.00345	0.00432	0.00432	0.949
rf	0.3284	0.00452	0.00362	0.00441	0.00450	0.952

Variable 9 mean = 0.3085

Method	mean	sd_Rubin	Absolute_bias	Simulated_sd	sqrt_MSE	Coverage
pmm	0.3089	0.00553	0.00435	0.00547	0.00548	0.950
sample	0.3089	0.00530	0.00448	0.00563	0.00565	0.935
cart	0.3093	0.00526	0.00438	0.00544	0.00548	0.941
rf	0.3061	0.00553	0.00488	0.00563	0.00611	0.921

1.4. Analysis of mean values - simulated MAR data

Variable 5 mean = 140

Method	mean	sd_Rubin	Absolute_bias	Simulated_sd	sqrt_MSE	Coverage
pmm	140.9	1.445	1.32	1.444	1.73	0.828
sample	132.3	0.241	7.73	0.307	7.73	0.000
cart	133.1	0.562	6.95	0.564	6.97	0.000
rf	132.0	0.538	8.03	0.345	8.04	0.000

Variable 6 mean = 110

Method	mean	sd_Rubin	Absolute_bias	Simulated_sd	sqrt_MSE	Coverage
pmm	110.0	0.236	0.192	0.237	0.237	0.949
sample	109.7	0.230	0.289	0.242	0.349	0.785
cart	110.1	0.230	0.203	0.237	0.253	0.925
rf	110.0	0.246	0.197	0.240	0.244	0.950

Variable 7 mean = 0.1587

Method	mean	sd_Rubin	Absolute_bias	Simulated_sd	sqrt_MSE	Coverage
pmm	0.1619	0.00591	0.00524	0.00563	0.00651	0.927
sample	0.1777	0.00487	0.01908	0.00595	0.01999	0.048
cart	0.1611	0.00543	0.00508	0.00588	0.00637	0.899
rf	0.1499	0.00617	0.00908	0.00593	0.01054	0.690

Variable 8 mean = 0.3293

Method	mean	sd_Rubin	Absolute_bias	Simulated_sd	sqrt_MSE	Coverage
pmm	0.3281	0.00440	0.00368	0.00441	0.00457	0.939
sample	0.3145	0.00418	0.01481	0.00437	0.01544	0.065
cart	0.3290	0.00425	0.00355	0.00441	0.00442	0.940
rf	0.3257	0.00490	0.00469	0.00449	0.00577	0.903

Variable 9 mean = 0.3085

Method	mean	sd_Rubin	Absolute_bias	Simulated_sd	sqrt_MSE	Coverage
pmm	0.3091	0.00548	0.00441	0.00555	0.00557	0.945
sample	0.3146	0.00532	0.00689	0.00565	0.00830	0.781
cart	0.3088	0.00524	0.00440	0.00554	0.00554	0.938
rf	0.3066	0.00574	0.00479	0.00567	0.00600	0.930

1.5. Analysis of mean values - simulated MNAR data

Variable 5 mean = 140

Method	mean	sd_Rubin	Absolute_bias	Simulated_sd	sqrt_MSE	Coverage
pmm	123.6	0.162	16.4	0.169	16.4	0
sample	120.7	0.142	19.3	0.176	19.3	0
cart	123.3	0.169	16.7	0.178	16.7	0
rf	122.0	0.263	18.0	0.171	18.1	0

Variable 6 mean = 110

Method	mean	sd_Rubin	Absolute_bias	Simulated_sd	sqrt_MSE	Coverage
pmm	108.2	0.237	1.84	0.237	1.86	0
sample	107.7	0.228	2.28	0.239	2.29	0
cart	108.1	0.230	1.89	0.238	1.90	0
rf	107.7	0.237	2.27	0.237	2.28	0

Variable 7 mean = 0.1587

Method	mean	sd_Rubin	Absolute_bias	Simulated_sd	sqrt_MSE	Coverage
pmm	0.3493	0.00746	0.191	0.00761	0.191	0
sample	0.3964	0.00625	0.238	0.00816	0.238	0
cart	0.3510	0.00664	0.192	0.00773	0.193	0
rf	0.3632	0.00765	0.205	0.00817	0.205	0

Variable 8 mean = 0.3293

Method	mean	sd_Rubin	Absolute_bias	Simulated_sd	sqrt_MSE	Coverage
pmm	0.2142	0.00431	0.115	0.00418	0.115	0
sample	0.1879	0.00369	0.141	0.00382	0.141	0

Method	mean	sd_Rubin	Absolute_bias	Simulated_sd	sqrt_MSE	Coverage
cart	0.2153	0.00405	0.114	0.00419	0.114	0
rf	0.2014	0.00439	0.128	0.00413	0.128	0

Variable 9 mean = 0.3085

Method	mean	sd_Rubin	Absolute_bias	Simulated_sd	sqrt_MSE	Coverage
pmm	0.3918	0.00578	0.0832	0.00603	0.0835	0
sample	0.4072	0.00563	0.0987	0.00614	0.0989	0
cart	0.3909	0.00553	0.0824	0.00599	0.0826	0
rf	0.3967	0.00639	0.0881	0.00618	0.0883	0

2. Variances and covariances

2.1. Analysis of variances and covariances - real data

Systolic blood pressure

Method	mean	inner	between	total
pmm	503.4	10.2	8.69	13.5
midastouch	504.5	10.4	13.51	17.3
sample	500.2	10.3	6.97	12.5
cart	518.2	10.6	7.79	13.2
rf	499.0	10.1	9.74	14.2

LDL

Method	mean	inner	between	total
pmm	1707	59.4	42.3	73.6
midastouch	1728	64.1	61.6	89.9
sample	1695	57.1	38.7	69.5
cart	1765	65.5	47.0	81.3
rf	1698	59.6	35.2	69.7

Diabetes

Method	mean	inner	between	total
pmm	0.2010	0.00231	0.00226	0.00327
midastouch	0.2009	0.00231	0.00303	0.00387
sample	0.1988	0.00235	0.00237	0.00337
cart	0.2036	0.00226	0.00224	0.00322
rf	0.1951	0.00241	0.00279	0.00374

Smoking behavior

Method	mean	inner	between	total
pmm	0.1617	0.00188	0.00113	0.00220
midastouch	0.1615	0.00188	0.00117	0.00223
sample	0.1592	0.00191	0.00114	0.00224
cart	0.1616	0.00186	0.00154	0.00244
rf	0.1591	0.00191	0.00157	0.00249

Family history of premature CAD

Method	mean	inner	between	total
pmm	0.1813	0.00259	0.00306	0.00407
midastouch	0.1818	0.00259	0.00144	0.00298
sample	0.1772	0.00264	0.00229	0.00353
cart	0.1841	0.00256	0.00189	0.00321
rf	0.1756	0.00266	0.00180	0.00324

2.2. Analysis of variances and covariances - simulated MCAR data

Variable 5 variance = 400.2

Method	mean	sd_Rubin	Absolute_bias	Simulated_sd	sqrt_MSE	Coverage
pmm	399.3	8.06	6.92	8.71	8.75	0.917
sample	399.4	7.21	7.31	9.23	9.26	0.872
cart	397.2	7.40	7.42	8.78	9.28	0.867
rf	385.6	7.95	14.95	8.64	16.97	0.545

Variable 6 variance = 399.9

Method	mean	sd_Rubin	Absolute_bias	Simulated_sd	sqrt_MSE	Coverage
pmm	400.4	6.66	5.30	6.67	6.68	0.942
sample	400.4	6.50	5.40	6.74	6.76	0.941
cart	400.0	6.54	5.30	6.66	6.66	0.945
rf	396.8	6.49	5.83	6.64	7.30	0.910

Variable 7 variance = 0.1337

Method	mean	sd_Rubin	Absolute_bias	Simulated_sd	sqrt_MSE	Coverage
pmm	0.1338	0.00399	0.00305	0.00387	0.00387	0.951
sample	0.1336	0.00318	0.00315	0.00401	0.00401	0.876
cart	0.1338	0.00338	0.00305	0.00385	0.00385	0.905
rf	0.1228	0.00375	0.01093	0.00409	0.01165	0.193

Variable 8 variance = 0.1356

Method	mean	sd_Rubin	Absolute_bias	Simulated_sd	sqrt_MSE	Coverage
pmm	0.1356	0.00165	0.00136	0.00169	0.00169	0.949
sample	0.1356	0.00162	0.00137	0.00170	0.00170	0.940
cart	0.1356	0.00162	0.00135	0.00169	0.00169	0.940
rf	0.1350	0.00166	0.00147	0.00170	0.00181	0.929

Variable 9 variance = 0.2134

Method	mean	sd_Rubin	Absolute_bias	Simulated_sd	sqrt_MSE	Coverage
pmm	0.2135	0.00211	0.00166	0.00209	0.00209	0.952
sample	0.2135	0.00203	0.00171	0.00216	0.00216	0.936
cart	0.2136	0.00201	0.00167	0.00208	0.00209	0.939
rf	0.2124	0.00214	0.00190	0.00218	0.00239	0.922

2.3. Analysis of variances and covariances - simulated MAR data

Variable 5 variance = 400.2

Method	mean	sd_Rubin	Absolute_bias	Simulated_sd	sqrt_MSE	Coverage
pmm	435.0	37.86	38.0	39.98	53.0	0.88
sample	356.1	6.43	44.1	7.84	44.8	0.00
cart	345.5	14.16	54.7	13.76	56.4	0.06

Method	mean	sd_Rubin	Absolute_bias	Simulated_sd	sqrt_MSE	Coverage
rf	340.5	8.50	59.7	7.91	60.2	0.00

Variable 6 variance = 399.9

Method	mean	sd_Rubin	Absolute_bias	Simulated_sd	sqrt_MSE	Coverage
pmm	401.3	6.70	5.48	6.73	6.89	0.950
sample	400.4	6.49	5.43	6.74	6.76	0.937
cart	397.2	6.55	5.84	6.80	7.30	0.921
rf	396.4	6.53	6.16	6.79	7.64	0.908

Variable 7 variance = 0.1337

Method	mean	sd_Rubin	Absolute_bias	Simulated_sd	sqrt_MSE	Coverage
pmm	0.1357	0.00400	0.00346	0.00381	0.00430	0.929
sample	0.1461	0.00313	0.01244	0.00384	0.01302	0.051
cart	0.1351	0.00368	0.00338	0.00398	0.00424	0.900
rf	0.1274	0.00431	0.00648	0.00415	0.00751	0.686

Variable 8 variance = 0.1356

Method	mean	sd_Rubin	Absolute_bias	Simulated_sd	sqrt_MSE	Coverage
pmm	0.1352	0.00168	0.00147	0.00178	0.00183	0.934
sample	0.1328	0.00165	0.00292	0.00177	0.00335	0.580
cart	0.1354	0.00166	0.00145	0.00179	0.00180	0.933
rf	0.1343	0.00173	0.00180	0.00181	0.00222	0.873

Variable 9 variance = 0.2134

Method	mean	sd_Rubin	Absolute_bias	Simulated_sd	sqrt_MSE	Coverage
pmm	0.2135	0.00209	0.00168	0.00212	0.00212	0.944
sample	0.2156	0.00197	0.00256	0.00210	0.00306	0.770
cart	0.2134	0.00200	0.00168	0.00212	0.00212	0.940
rf	0.2126	0.00222	0.00186	0.00220	0.00234	0.933

2.4. Analysis of variances and covariances - simulated MNAR data

Variable 5 variance = 400.2

Method	mean	sd_Rubin	Absolute_bias	Simulated_sd	sqrt_MSE	Coverage
pmm	95.56	2.45	305	2.44	305	0
sample	124.66	2.82	276	3.46	276	0
cart	97.36	2.65	303	2.67	303	0
rf	109.78	3.78	290	2.94	290	0

Variable 6 variance = 399.9

Method	mean	sd_Rubin	Absolute_bias	Simulated_sd	sqrt_MSE	Coverage
pmm	397.1	6.68	5.82	6.82	7.37	0.924
sample	394.8	6.50	6.88	6.77	8.47	0.860
cart	397.9	6.62	5.61	6.83	7.12	0.924
rf	391.9	6.46	8.77	6.71	10.45	0.749

Variable 7 variance = 0.1337

Method	mean	sd_Rubin	Absolute_bias	Simulated_sd	sqrt_MSE	Coverage
pmm	0.2272	0.00225	0.0935	0.00230	0.0936	0
sample	0.2392	0.00130	0.1055	0.00169	0.1056	0
cart	0.2278	0.00198	0.0941	0.00230	0.0941	0
rf	0.2312	0.00209	0.0975	0.00224	0.0976	0

Variable 8 variance = 0.1356

Method	mean	sd_Rubin	Absolute_bias	Simulated_sd	sqrt_MSE	Coverage
pmm	0.1155	0.00227	0.0201	0.00225	0.0203	0
sample	0.1038	0.00203	0.0318	0.00212	0.0319	0
cart	0.1159	0.00214	0.0197	0.00226	0.0198	0
rf	0.1095	0.00228	0.0261	0.00222	0.0262	0

Variable 9 variance = 0.2134

Method	mean	sd_Rubin	Absolute_bias	Simulated_sd	sqrt_MSE	Coverage
pmm	0.2383	0.00125	0.0249	0.00130	0.0249	0
sample	0.2414	0.00105	0.0280	0.00114	0.0280	0
cart	0.2381	0.00121	0.0247	0.00131	0.0247	0
rf	0.2393	0.00132	0.0259	0.00128	0.0259	0

3. Linear regression

3.1. Analysis of a linear regression - simulated MCAR data

Intercept = 100

Method	mean	sd_Rubin	Absolute_bias	Simulated_sd	sqrt_MSE	Coverage
pmm	99.91	1.92	1.58	1.964	1.97	0.943
sample	113.46	1.52	13.46	0.963	13.49	0.000
cart	100.03	1.81	1.75	2.163	2.16	0.895
rf	104.46	3.37	4.46	1.339	4.65	0.896

Variable 3 coefficient = 1

Method	mean	sd_Rubin	Absolute_bias	Simulated_sd	sqrt_MSE	Coverage
pmm	0.9962	0.0177	0.0141	0.0175	0.0179	0.938
sample	1.0807	0.0126	0.0807	0.0115	0.0815	0.000
cart	0.9908	0.0163	0.0157	0.0175	0.0197	0.886
rf	1.0021	0.0315	0.0110	0.0138	0.0139	0.998

Variable 4 coefficient = 20

Method	mean	sd_Rubin	Absolute_bias	Simulated_sd	sqrt_MSE	Coverage
pmm	19.94	0.348	0.282	0.348	0.352	0.954
sample	17.26	0.343	2.742	0.307	2.759	0.000
cart	19.54	0.353	0.498	0.383	0.595	0.727
rf	18.62	0.442	1.377	0.322	1.414	0.061

Variable 5 coefficient = 0.2

Method	mean	sd_Rubin	Absolute_bias	Simulated_sd	sqrt_MSE	Coverage
pmm	0.20254	0.01206	0.00992	0.01219	0.0124	0.933
sample	0.08201	0.00789	0.11799	0.00353	0.1180	0.000
cart	0.20245	0.01106	0.01113	0.01389	0.0141	0.847
rf	0.17460	0.02578	0.02541	0.00816	0.0267	0.992

Variable 6 coefficient = -0.2

Method	mean	sd_Rubin	Absolute_bias	Simulated_sd	sqrt_MSE	Coverage
pmm	-0.1998	0.00972	0.00785	0.00990	0.00990	0.939
sample	-0.2099	0.00736	0.00998	0.00468	0.01098	0.838
cart	-0.1963	0.00893	0.00808	0.00941	0.01010	0.912
rf	-0.2070	0.01469	0.00782	0.00626	0.00939	0.998

Variable 7 coefficient = 5

Method	mean	sd_Rubin	Absolute_bias	Simulated_sd	sqrt_MSE	Coverage
pmm	5.097	0.539	0.422	0.515	0.524	0.955
sample	2.110	0.416	2.890	0.202	2.897	0.000
cart	4.117	0.511	0.897	0.490	1.010	0.584
rf	2.681	0.546	2.319	0.329	2.342	0.000

Variable 8 coefficient = -10

Method	mean	sd_Rubin	Absolute_bias	Simulated_sd	sqrt_MSE	Coverage
pmm	-10.075	0.450	0.362	0.449	0.455	0.947
sample	-5.736	0.388	4.264	0.271	4.272	0.000
cart	-9.136	0.445	0.875	0.453	0.975	0.491
rf	-6.366	0.491	3.634	0.301	3.646	0.000

Variable 9 coefficient = 1

Method	mean	sd_Rubin	Absolute_bias	Simulated_sd	sqrt_MSE	Coverage
pmm	1.064	0.332	0.270	0.333	0.338	0.940
sample	1.874	0.303	0.874	0.205	0.897	0.079
cart	1.192	0.313	0.281	0.294	0.352	0.921
rf	1.528	0.328	0.529	0.229	0.575	0.705

3.2. Analysis of a linear regression - simulated MAR data

Intercept = 100

Method	mean	sd_Rubin	Absolute_bias	Simulated_sd	sqrt_MSE	Coverage
pmm	100.1	1.99	1.62	2.02	2.02	0.952
sample	111.1	1.59	11.07	1.02	11.12	0.000
cart	102.8	3.26	3.12	2.56	3.76	0.918
rf	105.6	3.70	5.60	1.43	5.78	0.804

Variable 3 coefficient = 1

Method	mean	sd_Rubin	Absolute_bias	Simulated_sd	sqrt_MSE	Coverage
pmm	0.9943	0.0196	0.0165	0.0201	0.0209	0.933
sample	1.0974	0.0123	0.0974	0.0112	0.0981	0.000
cart	1.1019	0.0219	0.1019	0.0192	0.1037	0.008
rf	1.0806	0.0240	0.0806	0.0127	0.0816	0.017

Variable 4 coefficient = 20

Method	mean	sd_Rubin	Absolute_bias	Simulated_sd	sqrt_MSE	Coverage
pmm	19.87	0.340	0.277	0.322	0.346	0.947
sample	17.78	0.339	2.215	0.292	2.235	0.000
cart	19.05	0.388	0.950	0.361	1.016	0.297
rf	18.42	0.401	1.584	0.298	1.611	0.002

Variable 5 coefficient = 0.2

Method	mean	sd_Rubin	Absolute_bias	Simulated_sd	sqrt_MSE	Coverage
pmm	0.20262	0.01253	0.0103	0.01267	0.0129	0.939
sample	0.09004	0.00826	0.1100	0.00396	0.1100	0.000
cart	0.15502	0.02130	0.0451	0.01708	0.0481	0.407
rf	0.14405	0.02523	0.0560	0.00863	0.0566	0.291

Variable 6 coefficient = -0.2

Method	mean	sd_Rubin	Absolute_bias	Simulated_sd	sqrt_MSE	Coverage
pmm	-0.2014	0.01000	0.00825	0.01034	0.01042	0.937
sample	-0.2077	0.00738	0.00793	0.00481	0.00911	0.914

Method	mean	sd_Rubin	Absolute_bias	Simulated_sd	sqrt_MSE	Coverage
cart	-0.2188	0.01357	0.01917	0.01115	0.02184	0.756
rf	-0.2176	0.01528	0.01765	0.00647	0.01878	0.940

Variable 7 coefficient = 5

Method	mean	sd_Rubin	Absolute_bias	Simulated_sd	sqrt_MSE	Coverage
pmm	5.008	0.509	0.412	0.514	0.514	0.938
sample	1.970	0.398	3.030	0.198	3.036	0.000
cart	3.751	0.575	1.252	0.484	1.339	0.396
rf	2.577	0.535	2.423	0.328	2.445	0.000

Variable 8 coefficient = -10

Method	mean	sd_Rubin	Absolute_bias	Simulated_sd	sqrt_MSE	Coverage
pmm	-9.803	0.458	0.409	0.470	0.509	0.920
sample	-5.737	0.396	4.263	0.271	4.271	0.000
cart	-8.394	0.509	1.606	0.464	1.671	0.116
rf	-6.115	0.508	3.885	0.305	3.897	0.000

Variable 9 coefficient = 1

Method	mean	sd_Rubin	Absolute_bias	Simulated_sd	sqrt_MSE	Coverage
pmm	1.126	0.329	0.279	0.324	0.347	0.936
sample	1.956	0.301	0.956	0.205	0.977	0.041
cart	1.265	0.348	0.333	0.310	0.408	0.914
rf	1.516	0.348	0.518	0.234	0.566	0.755

3.3. Analysis of a linear regression - simulated MNAR data

Intercept = 100

Method	mean	sd_Rubin	Absolute_bias	Simulated_sd	sqrt_MSE	Coverage
pmm	104.8	2.61	4.84	2.46	5.39	0.549
sample	113.5	1.96	13.46	1.14	13.51	0.000
cart	105.8	2.63	5.85	2.76	6.44	0.415
rf	105.1	3.66	5.06	1.72	5.35	0.838

Variable 3 coefficient = 1

Method	mean	sd_Rubin	Absolute_bias	Simulated_sd	sqrt_MSE	Coverage
pmm	1.168	0.0145	0.168	0.0139	0.168	0
sample	1.104	0.0125	0.104	0.0110	0.105	0
cart	1.161	0.0140	0.161	0.0138	0.161	0
rf	1.101	0.0170	0.101	0.0115	0.101	0

Variable 4 coefficient = 20

Method	mean	sd_Rubin	Absolute_bias	Simulated_sd	sqrt_MSE	Coverage
pmm	18.35	0.348	1.65	0.333	1.69	0.001
sample	16.79	0.343	3.21	0.306	3.22	0.000
cart	17.74	0.349	2.26	0.340	2.29	0.000
rf	17.14	0.371	2.86	0.311	2.87	0.000

Variable 5 coefficient = 0.2

Method	mean	sd_Rubin	Absolute_bias	Simulated_sd	sqrt_MSE	Coverage
pmm	0.16232	0.0197	0.0380	0.01822	0.0419	0.518
sample	0.07785	0.0137	0.1221	0.00617	0.1223	0.000
cart	0.15702	0.0201	0.0433	0.02112	0.0479	0.440
rf	0.16786	0.0288	0.0322	0.01230	0.0344	0.922

Variable 6 coefficient = -0.2

Method	mean	sd_Rubin	Absolute_bias	Simulated_sd	sqrt_MSE	Coverage
pmm	-0.2832	0.00782	0.0832	0.00785	0.0835	0.000
sample	-0.2200	0.00734	0.0200	0.00491	0.0206	0.126
cart	-0.2777	0.00764	0.0777	0.00778	0.0781	0.000
rf	-0.2440	0.01164	0.0440	0.00612	0.0444	0.000

Variable 7 coefficient = 5

Method	mean	sd_Rubin	Absolute_bias	Simulated_sd	sqrt_MSE	Coverage
pmm	6.511	0.424	1.511	0.414	1.567	0.06
sample	2.128	0.312	2.872	0.160	2.876	0.00
cart	5.261	0.417	0.384	0.396	0.474	0.91
rf	2.879	0.413	2.121	0.250	2.135	0.00

Variable 8 coefficient = -10

Method	mean	sd_Rubin	Absolute_bias	Simulated_sd	sqrt_MSE	Coverage
pmm	-9.841	0.510	0.422	0.505	0.529	0.942
sample	-5.708	0.442	4.292	0.299	4.302	0.000
cart	-8.670	0.507	1.331	0.501	1.422	0.253
rf	-5.926	0.533	4.074	0.332	4.088	0.000

Variable 9 coefficient = 1

Method	mean	sd_Rubin	Absolute_bias	Simulated_sd	sqrt_MSE	Coverage
pmm	0.3138	0.325	0.691	0.319	0.756	0.430
sample	1.9427	0.288	0.943	0.196	0.963	0.028
cart	0.7671	0.312	0.310	0.307	0.385	0.892
rf	1.4688	0.322	0.472	0.227	0.521	0.759

4. Logistic regression

4.1. Analysis of the coefficients of a logistic regression model - real data

Intercept

Method	mean	inner	between	total
pmm	-1.525	0.310	0.285	0.426
midastouch	-1.860	0.311	0.249	0.402
sample	-2.732	0.307	0.168	0.352
cart	-1.946	0.308	0.299	0.434
rf	-1.854	0.309	0.214	0.379

Age

Method	mean	inner	between	total
pmm	0.03108	0.00312	0.001282	0.00339
midastouch	0.03037	0.00310	0.001618	0.00352
sample	0.02540	0.00293	0.000425	0.00296
cart	0.03002	0.00311	0.001100	0.00331
rf	0.02670	0.00302	0.001091	0.00322

Sex

Method	mean	inner	between	total
pmm	0.1121	0.0764	0.02332	0.0801
midastouch	0.1174	0.0761	0.01894	0.0785
sample	0.1560	0.0750	0.00545	0.0752
cart	0.1126	0.0761	0.01657	0.0780
rf	0.1160	0.0758	0.01452	0.0772

Systolic blood pressure

Method	mean	inner	between	total
pmm	-0.015604	0.00153	0.002272	0.00278
midastouch	-0.012923	0.00150	0.002185	0.00270
sample	-0.004309	0.00143	0.000813	0.00166
cart	-0.012194	0.00149	0.001947	0.00249
rf	-0.010175	0.00147	0.001554	0.00217

LDL

Method	mean	inner	between	total
pmm	-0.003314	0.000840	0.001146	0.00144
midastouch	-0.002950	0.000833	0.000937	0.00127
sample	-0.001781	0.000799	0.000654	0.00104
cart	-0.002942	0.000829	0.001072	0.00138
rf	-0.003504	0.000827	0.000933	0.00126

Diabetes

Method	mean	inner	between	total
pmm	0.3451	0.0690	0.0798	0.1070

Method	mean	inner	between	total
midastouch	0.2885	0.0688	0.0888	0.1141
sample	0.1092	0.0695	0.0559	0.0901
cart	0.3204	0.0686	0.0941	0.1183
rf	0.2639	0.0690	0.0645	0.0955

Smoking behavior

Method	mean	inner	between	total
pmm	0.5610	0.0836	0.0630	0.1057
midastouch	0.5475	0.0835	0.1062	0.1371
sample	0.3026	0.0801	0.0475	0.0937
cart	0.5396	0.0835	0.0588	0.1030
rf	0.4226	0.0827	0.0713	0.1103

Family history of premature CAD

Method	mean	inner	between	total
pmm	-0.2045	0.0813	0.0610	0.1025
midastouch	-0.1970	0.0810	0.0703	0.1084
sample	-0.1156	0.0778	0.0517	0.0941
cart	-0.1701	0.0802	0.0604	0.1013
rf	-0.2688	0.0818	0.0535	0.0985

4.2. Analysis of a logistic regression - simulated MCAR data

Intercept = -5

Method	mean	sd_Rubin	Absolute_bias	Simulated_sd	sqrt_MSE	Coverage
pmm	-5.021	0.447	0.361	0.453	0.454	0.948
sample	-4.261	0.285	0.739	0.209	0.768	0.207
cart	-4.623	0.371	0.416	0.328	0.499	0.854
rf	-5.286	0.425	0.351	0.319	0.428	0.963

Variable 3 coefficient = 0.05

Method	mean	sd_Rubin	Absolute_bias	Simulated_sd	sqrt_MSE	Coverage
pmm	0.04983	0.00394	0.00312	0.00391	0.00391	0.949
sample	0.05311	0.00244	0.00334	0.00245	0.00396	0.763
cart	0.05174	0.00341	0.00285	0.00307	0.00353	0.944
rf	0.04901	0.00298	0.00217	0.00254	0.00273	0.972

Variable 4 coefficient = 1

Method	mean	sd_Rubin	Absolute_bias	Simulated_sd	sqrt_MSE	Coverage
pmm	1.0001	0.0784	0.0625	0.0787	0.0786	0.953
sample	0.8554	0.0705	0.1456	0.0691	0.1603	0.445
cart	0.9442	0.0756	0.0765	0.0748	0.0933	0.888
rf	0.9615	0.0773	0.0665	0.0730	0.0825	0.933

Variable 5 coefficient = 0.01

Method	mean	sd_Rubin	Absolute_bias	Simulated_sd	sqrt_MSE	Coverage
pmm	0.010163	0.00283	0.00222	0.002834	0.00284	0.934
sample	0.004009	0.00146	0.00599	0.000698	0.00603	0.000
cart	0.007038	0.00225	0.00308	0.001841	0.00349	0.777
rf	0.012899	0.00268	0.00296	0.001793	0.00341	0.906

Variable 6 coefficient = -0.01

Method	mean	sd_Rubin	Absolute_bias	Simulated_sd	sqrt_MSE	Coverage
pmm	-0.009953	0.00219	0.001730	0.002176	0.002175	0.943
sample	-0.010277	0.00133	0.000783	0.000932	0.000972	0.994
cart	-0.010482	0.00188	0.001360	0.001619	0.001688	0.973
rf	-0.010535	0.00162	0.001128	0.001298	0.001404	0.981

Variable 7 coefficient = 0.25

Method	mean	sd_Rubin	Absolute_bias	Simulated_sd	sqrt_MSE	Coverage
pmm	0.2496	0.1158	0.0951	0.1183	0.1182	0.941
sample	0.1027	0.0779	0.1473	0.0391	0.1524	0.553
cart	0.1856	0.0975	0.0814	0.0762	0.0997	0.941
rf	0.1256	0.0910	0.1274	0.0773	0.1465	0.751

Variable 8 coefficient = -0.5

Method	mean	sd_Rubin	Absolute_bias	Simulated_sd	sqrt_MSE	Coverage
pmm	-0.4981	0.0960	0.074	0.0910	0.091	0.962
sample	-0.2811	0.0720	0.219	0.0499	0.224	0.066
cart	-0.3933	0.0888	0.114	0.0784	0.132	0.792
rf	-0.3500	0.0841	0.151	0.0649	0.163	0.563

Variable 9 coefficient = 0.05

Method	mean	sd_Rubin	Absolute_bias	Simulated_sd	sqrt_MSE	Coverage
pmm	0.05546	0.0659	0.0511	0.0648	0.0650	0.947
sample	0.09217	0.0555	0.0475	0.0382	0.0569	0.959
cart	0.06453	0.0610	0.0423	0.0518	0.0537	0.978
rf	0.08471	0.0599	0.0481	0.0490	0.0601	0.952

4.3. Analysis of a logistic regression - simulated MAR data

Intercept = -5

Method	mean	sd_Rubin	Absolute_bias	Simulated_sd	sqrt_MSE	Coverage
pmm	-4.994	0.475	0.381	0.477	0.477	0.947
sample	-4.298	0.303	0.702	0.201	0.730	0.292
cart	-4.779	0.685	0.433	0.493	0.540	0.978
rf	-5.101	0.547	0.291	0.351	0.365	0.997

Variable 3 coefficient = 0.05

Method	mean	sd_Rubin	Absolute_bias	Simulated_sd	sqrt_MSE	Coverage
pmm	0.04948	0.00418	0.00339	0.00426	0.00429	0.941
sample	0.05392	0.00239	0.00401	0.00241	0.00460	0.607

Method	mean	sd_Rubin	Absolute_bias	Simulated_sd	sqrt_MSE	Coverage
cart	0.05605	0.00282	0.00607	0.00263	0.00659	0.415
rf	0.05526	0.00254	0.00529	0.00246	0.00581	0.449

Variable 4 coefficient = 1

Method	mean	sd_Rubin	Absolute_bias	Simulated_sd	sqrt_MSE	Coverage
pmm	0.9926	0.0786	0.0608	0.0763	0.0767	0.949
sample	0.8679	0.0707	0.1337	0.0683	0.1487	0.538
cart	0.9314	0.0798	0.0839	0.0743	0.1011	0.869
rf	0.9409	0.0777	0.0758	0.0711	0.0924	0.902

Variable 5 coefficient = 0.01

Method	mean	sd_Rubin	Absolute_bias	Simulated_sd	sqrt_MSE	Coverage
pmm	0.010136	0.00307	0.00250	0.003125	0.00313	0.937
sample	0.003805	0.00156	0.00620	0.000689	0.00623	0.000
cart	0.006281	0.00376	0.00402	0.002614	0.00455	0.824
rf	0.009822	0.00322	0.00152	0.001886	0.00189	0.994

Variable 6 coefficient = -0.01

Method	mean	sd_Rubin	Absolute_bias	Simulated_sd	sqrt_MSE	Coverage
pmm	-0.009948	0.00229	0.001883	0.002361	0.002360	0.940
sample	-0.010076	0.00133	0.000743	0.000944	0.000946	0.990
cart	-0.010252	0.00242	0.001494	0.001865	0.001881	0.981
rf	-0.011233	0.00171	0.001514	0.001397	0.001863	0.934

Variable 7 coefficient = 0.25

Method	mean	sd_Rubin	Absolute_bias	Simulated_sd	sqrt_MSE	Coverage
pmm	0.23837	0.1141	0.0899	0.1111	0.112	0.947
sample	0.08959	0.0744	0.1604	0.0346	0.164	0.336
cart	0.17773	0.1048	0.0855	0.0734	0.103	0.968
rf	0.09936	0.0935	0.1516	0.0720	0.167	0.669

Variable 8 coefficient = -0.5

Method	mean	sd_Rubin	Absolute_bias	Simulated_sd	sqrt_MSE	Coverage
pmm	-0.4862	0.0983	0.0769	0.0965	0.0974	0.949
sample	-0.2754	0.0732	0.2246	0.0497	0.2300	0.052
cart	-0.3874	0.0928	0.1189	0.0815	0.1389	0.778
rf	-0.3288	0.0865	0.1713	0.0665	0.1837	0.487

Variable 9 coefficient = 0.05

Method	mean	sd_Rubin	Absolute_bias	Simulated_sd	sqrt_MSE	Coverage
pmm	0.05967	0.0657	0.0539	0.0676	0.0682	0.937
sample	0.09903	0.0548	0.0535	0.0400	0.0633	0.928
cart	0.07245	0.0623	0.0472	0.0548	0.0592	0.958
rf	0.08359	0.0598	0.0495	0.0518	0.0618	0.947

4.4. Analysis of a logistic regression - simulated MNAR data

Intercept = -5

Method	mean	sd_Rubin	Absolute_bias	Simulated_sd	sqrt_MSE	Coverage
pmm	-4.618	0.523	0.500	0.483	0.615	0.890
sample	-4.137	0.374	0.863	0.225	0.892	0.297
cart	-4.470	0.504	0.580	0.436	0.686	0.838
rf	-5.162	0.554	0.377	0.441	0.470	0.985

Variable 3 coefficient = 0.05

Method	mean	sd_Rubin	Absolute_bias	Simulated_sd	sqrt_MSE	Coverage
pmm	0.05906	0.00289	0.00906	0.00289	0.00951	0.123
sample	0.05423	0.00240	0.00429	0.00240	0.00486	0.565
cart	0.05804	0.00276	0.00804	0.00273	0.00849	0.166
rf	0.05492	0.00257	0.00496	0.00248	0.00551	0.499

Variable 4 coefficient = 1

Method	mean	sd_Rubin	Absolute_bias	Simulated_sd	sqrt_MSE	Coverage
pmm	0.9060	0.0752	0.103	0.0748	0.120	0.775
sample	0.8258	0.0701	0.174	0.0690	0.187	0.299
cart	0.8741	0.0744	0.129	0.0729	0.145	0.603
rf	0.8569	0.0735	0.145	0.0715	0.160	0.497

Variable 5 coefficient = 0.01

Method	mean	sd_Rubin	Absolute_bias	Simulated_sd	sqrt_MSE	Coverage
pmm	0.006881	0.00393	0.00390	0.00359	0.00476	0.887
sample	0.002893	0.00262	0.00711	0.00115	0.00720	0.050
cart	0.006089	0.00383	0.00431	0.00319	0.00505	0.848
rf	0.012530	0.00427	0.00335	0.00330	0.00416	0.971

Variable 6 coefficient = -0.01

Method	mean	sd_Rubin	Absolute_bias	Simulated_sd	sqrt_MSE	Coverage
pmm	-0.01449	0.00160	0.00450	0.001572	0.00476	0.188
sample	-0.01081	0.00132	0.00102	0.000944	0.00124	0.971
cart	-0.01406	0.00152	0.00407	0.001584	0.00436	0.245
rf	-0.01289	0.00150	0.00291	0.001348	0.00319	0.510

Variable 7 coefficient = 0.25

Method	mean	sd_Rubin	Absolute_bias	Simulated_sd	sqrt_MSE	Coverage
pmm	0.3404	0.0887	0.1020	0.0869	0.1253	0.836
sample	0.1022	0.0578	0.1478	0.0285	0.1505	0.119
cart	0.2381	0.0797	0.0576	0.0701	0.0711	0.974
rf	0.1572	0.0691	0.0956	0.0551	0.1079	0.768

Variable 8 coefficient = -0.5

Method	mean	sd_Rubin	Absolute_bias	Simulated_sd	sqrt_MSE	Coverage
pmm	-0.4928	0.1087	0.0882	0.1095	0.110	0.942
sample	-0.2784	0.0816	0.2217	0.0583	0.229	0.156
cart	-0.3783	0.1030	0.1313	0.0956	0.155	0.774

Method	mean	sd_Rubin	Absolute_bias	Simulated_sd	sqrt_MSE	Coverage
rf	-0.3244	0.0950	0.1764	0.0748	0.191	0.545

Variable 9 coefficient = 0.05

Method	mean	sd_Rubin	Absolute_bias	Simulated_sd	sqrt_MSE	Coverage
pmm	0.01200	0.0631	0.0587	0.0630	0.0736	0.917
sample	0.09809	0.0524	0.0514	0.0372	0.0608	0.924
cart	0.04270	0.0599	0.0425	0.0536	0.0540	0.968
rf	0.07895	0.0578	0.0440	0.0474	0.0556	0.954

5. Cox regression

5.1. Analysis of the coefficients of a Cox regression model - real data

Age

Method	mean	inner	between	total
pmm	0.03700	0.00294	0.000985	0.00311
midastouch	0.03658	0.00293	0.001163	0.00316
sample	0.03399	0.00282	0.000470	0.00286
cart	0.03593	0.00293	0.001274	0.00321
rf	0.03464	0.00289	0.000972	0.00305

Sex

Method	mean	inner	between	total
pmm	-0.007359	0.0688	0.01848	0.0714
midastouch	-0.005486	0.0687	0.01764	0.0711
sample	0.038361	0.0683	0.00693	0.0687
cart	-0.005857	0.0689	0.02649	0.0741
rf	-0.010028	0.0688	0.01969	0.0717

Systolic blood pressure

Method	mean	inner	between	total
pmm	-0.013069	0.00136	0.002478	0.00288
midastouch	-0.011304	0.00135	0.001990	0.00245
sample	-0.003843	0.00131	0.000768	0.00153
cart	-0.012932	0.00137	0.002284	0.00271
rf	-0.010964	0.00136	0.002220	0.00265

LDL

Method	mean	inner	between	total
pmm	-0.0021806	0.000772	0.000747	0.00109
midastouch	-0.0019992	0.000762	0.000886	0.00119
sample	-0.0009722	0.000737	0.000569	0.00094
cart	-0.0014633	0.000759	0.001172	0.00142
rf	-0.0024073	0.000766	0.000813	0.00113

Diabetes

Method	mean	inner	between	total
pmm	0.3775	0.0612	0.0921	0.1125
midastouch	0.2971	0.0615	0.0549	0.0833
sample	0.1325	0.0628	0.0513	0.0819
cart	0.4730	0.0603	0.0761	0.0986
rf	0.3180	0.0620	0.0683	0.0935

Smoking behavior

Method	mean	inner	between	total
pmm	0.4728	0.0744	0.0700	0.1033

Method	mean	inner	between	total
midastouch	0.4544	0.0742	0.0620	0.0977
sample	0.2402	0.0726	0.0528	0.0906
cart	0.4327	0.0749	0.0681	0.1024
rf	0.3718	0.0745	0.0629	0.0985

Family history of premature CAD

Method	mean	inner	between	total
pmm	-0.5032	0.0752	0.0724	0.1056
midastouch	-0.4964	0.0749	0.0425	0.0867
sample	-0.2196	0.0712	0.0519	0.0889
cart	-0.4519	0.0732	0.0680	0.1010
rf	-0.4784	0.0766	0.0514	0.0930

5.2. Analysis of a Cox regression - simulated MCAR data

Variable 3 coefficient = 0.05

Method	mean	sd_Rubin	Absolute_bias	Simulated_sd	sqrt_MSE	Coverage
pmm	0.04948	0.00294	0.00239	0.00291	0.00296	0.955
sample	0.05247	0.00187	0.00259	0.00178	0.00304	0.749
cart	0.05164	0.00259	0.00228	0.00236	0.00288	0.919
rf	0.04939	0.00227	0.00158	0.00188	0.00197	0.972

Variable 4 coefficient = 1

Method	mean	sd_Rubin	Absolute_bias	Simulated_sd	sqrt_MSE	Coverage
pmm	0.9715	0.0500	0.0454	0.0488	0.0565	0.916
sample	0.8350	0.0430	0.1650	0.0413	0.1701	0.024
cart	0.9264	0.0478	0.0756	0.0455	0.0865	0.656
rf	0.9194	0.0488	0.0817	0.0434	0.0916	0.623

Variable 5 coefficient = 0.01

Method	mean	sd_Rubin	Absolute_bias	Simulated_sd	sqrt_MSE	Coverage
pmm	0.009321	0.00207	0.001701	0.002013	0.00212	0.936
sample	0.004028	0.00113	0.005972	0.000517	0.00599	0.000
cart	0.006916	0.00170	0.003108	0.001431	0.00340	0.550
rf	0.010353	0.00176	0.000946	0.001121	0.00117	0.997

Variable 6 coefficient = -0.01

Method	mean	sd_Rubin	Absolute_bias	Simulated_sd	sqrt_MSE	Coverage
pmm	-0.009858	0.00163	0.001265	0.001583	0.001589	0.947
sample	-0.010156	0.00103	0.000572	0.000694	0.000711	0.996
cart	-0.010642	0.00142	0.001140	0.001256	0.001409	0.950
rf	-0.010314	0.00122	0.000783	0.000923	0.000975	0.985

Variable 7 coefficient = 0.25

Method	mean	sd_Rubin	Absolute_bias	Simulated_sd	sqrt_MSE	Coverage
pmm	0.2323	0.0859	0.0678	0.0826	0.0844	0.956

Method	mean	sd_Rubin	Absolute_bias	Simulated_sd	sqrt_MSE	Coverage
sample	0.1045	0.0598	0.1455	0.0312	0.1488	0.182
cart	0.1840	0.0736	0.0742	0.0596	0.0889	0.905
rf	0.1507	0.0703	0.1015	0.0566	0.1143	0.748

Variable 8 coefficient = -0.5

Method	mean	sd_Rubin	Absolute_bias	Simulated_sd	sqrt_MSE	Coverage
pmm	-0.4734	0.0720	0.0581	0.0677	0.0727	0.943
sample	-0.2737	0.0552	0.2263	0.0371	0.2293	0.000
cart	-0.3922	0.0676	0.1098	0.0599	0.1233	0.642
rf	-0.3336	0.0638	0.1664	0.0468	0.1728	0.191

Variable 9 coefficient = 0.05

Method	mean	sd_Rubin	Absolute_bias	Simulated_sd	sqrt_MSE	Coverage
pmm	0.05505	0.0528	0.0412	0.0513	0.0515	0.948
sample	0.09202	0.0447	0.0450	0.0315	0.0525	0.931
cart	0.06611	0.0495	0.0377	0.0443	0.0471	0.957
rf	0.08633	0.0487	0.0444	0.0399	0.0540	0.935

5.3. Analysis of a Cox regression - simulated MAR data

Variable 3 coefficient = 0.05

Method	mean	sd_Rubin	Absolute_bias	Simulated_sd	sqrt_MSE	Coverage
pmm	0.04759	0.00294	0.00311	0.00295	0.00381	0.860
sample	0.05334	0.00182	0.00337	0.00181	0.00380	0.560
cart	0.05473	0.00230	0.00475	0.00212	0.00519	0.464
rf	0.05347	0.00200	0.00349	0.00185	0.00393	0.600

Variable 4 coefficient = 1

Method	mean	sd_Rubin	Absolute_bias	Simulated_sd	sqrt_MSE	Coverage
pmm	0.9974	0.0492	0.0380	0.0482	0.0482	0.951
sample	0.8886	0.0435	0.1115	0.0415	0.1188	0.251
cart	0.9448	0.0499	0.0613	0.0473	0.0727	0.811
rf	0.9410	0.0485	0.0627	0.0434	0.0732	0.807

Variable 5 coefficient = 0.01

Method	mean	sd_Rubin	Absolute_bias	Simulated_sd	sqrt_MSE	Coverage
pmm	0.011451	0.00198	0.00199	0.001998	0.00247	0.884
sample	0.005687	0.00115	0.00431	0.000648	0.00436	0.001
cart	0.007781	0.00232	0.00242	0.001740	0.00282	0.886
rf	0.009674	0.00198	0.00101	0.001220	0.00126	0.996

Variable 6 coefficient = -0.01

Method	mean	sd_Rubin	Absolute_bias	Simulated_sd	sqrt_MSE	Coverage
pmm	-0.009093	0.00158	0.001459	0.001554	0.001798	0.915
sample	-0.010032	0.00104	0.000575	0.000717	0.000717	0.995
cart	-0.010290	0.00162	0.001067	0.001296	0.001328	0.982

Method	mean	sd_Rubin	Absolute_bias	Simulated_sd	sqrt_MSE	Coverage
rf	-0.010411	0.00130	0.000820	0.000943	0.001028	0.987

Variable 7 coefficient = 0.25

Method	mean	sd_Rubin	Absolute_bias	Simulated_sd	sqrt_MSE	Coverage
pmm	0.2221	0.0809	0.0664	0.0783	0.0831	0.936
sample	0.1042	0.0568	0.1458	0.0300	0.1488	0.131
cart	0.1780	0.0741	0.0778	0.0569	0.0918	0.894
rf	0.1410	0.0696	0.1101	0.0529	0.1211	0.692

Variable 8 coefficient = -0.5

Method	mean	sd_Rubin	Absolute_bias	Simulated_sd	sqrt_MSE	Coverage
pmm	-0.4839	0.0716	0.0561	0.0692	0.071	0.948
sample	-0.2892	0.0564	0.2108	0.0390	0.214	0.005
cart	-0.3972	0.0690	0.1058	0.0610	0.119	0.696
rf	-0.3371	0.0646	0.1629	0.0483	0.170	0.236

Variable 9 coefficient = 0.05

Method	mean	sd_Rubin	Absolute_bias	Simulated_sd	sqrt_MSE	Coverage
pmm	0.06112	0.0522	0.0418	0.0513	0.0524	0.950
sample	0.09292	0.0442	0.0457	0.0318	0.0534	0.917
cart	0.06991	0.0500	0.0393	0.0448	0.0490	0.956
rf	0.08494	0.0489	0.0442	0.0409	0.0538	0.935

5.4. Analysis of a Cox regression - simulated MNAR data

Variable 3 coefficient = 0.05

Method	mean	sd_Rubin	Absolute_bias	Simulated_sd	sqrt_MSE	Coverage
pmm	0.05645	0.00218	0.00645	0.00214	0.00679	0.158
sample	0.05351	0.00184	0.00353	0.00183	0.00396	0.533
cart	0.05645	0.00213	0.00645	0.00211	0.00678	0.149
rf	0.05354	0.00199	0.00357	0.00188	0.00401	0.583

Variable 4 coefficient = 1

Method	mean	sd_Rubin	Absolute_bias	Simulated_sd	sqrt_MSE	Coverage
pmm	0.8959	0.0475	0.104	0.0460	0.114	0.418
sample	0.8149	0.0428	0.185	0.0417	0.190	0.007
cart	0.8640	0.0468	0.136	0.0451	0.143	0.159
rf	0.8429	0.0462	0.157	0.0431	0.163	0.050

Variable 5 coefficient = 0.01

Method	mean	sd_Rubin	Absolute_bias	Simulated_sd	sqrt_MSE	Coverage
pmm	0.008584	0.00250	0.00213	0.002294	0.00269	0.918
sample	0.005028	0.00187	0.00497	0.000974	0.00507	0.096
cart	0.007471	0.00243	0.00275	0.002085	0.00328	0.855
rf	0.011089	0.00247	0.00172	0.001826	0.00213	0.979

Variable 6 coefficient = -0.01

Method	mean	sd_Rubin	Absolute_bias	Simulated_sd	sqrt_MSE	Coverage
pmm	-0.01319	0.00122	0.003193	0.001160	0.00340	0.238
sample	-0.01051	0.00102	0.000717	0.000718	0.00088	0.977
cart	-0.01305	0.00117	0.003056	0.001153	0.00326	0.253
rf	-0.01175	0.00113	0.001768	0.000921	0.00198	0.711

Variable 7 coefficient = 0.25

Method	mean	sd_Rubin	Absolute_bias	Simulated_sd	sqrt_MSE	Coverage
pmm	0.2924	0.0652	0.0634	0.0654	0.0780	0.900
sample	0.1088	0.0447	0.1412	0.0239	0.1432	0.011
cart	0.2196	0.0587	0.0488	0.0535	0.0615	0.930
rf	0.1545	0.0528	0.0957	0.0423	0.1044	0.569

Variable 8 coefficient = -0.5

Method	mean	sd_Rubin	Absolute_bias	Simulated_sd	sqrt_MSE	Coverage
pmm	-0.4597	0.0794	0.0691	0.0771	0.087	0.927
sample	-0.2776	0.0621	0.2224	0.0439	0.227	0.017
cart	-0.3763	0.0757	0.1264	0.0699	0.142	0.625
rf	-0.3153	0.0709	0.1847	0.0524	0.192	0.191

Variable 9 coefficient = 0.05

Method	mean	sd_Rubin	Absolute_bias	Simulated_sd	sqrt_MSE	Coverage
pmm	0.02395	0.0496	0.0445	0.0499	0.0563	0.915
sample	0.09112	0.0417	0.0437	0.0304	0.0511	0.912
cart	0.05321	0.0473	0.0352	0.0440	0.0441	0.961
rf	0.08237	0.0467	0.0411	0.0383	0.0502	0.944

6. The txt-files

The tables are also supplied in *.txt-files so that the reader can easily build further analysis based on them, e.g. the meta-analyses proposed in the discussion of the main article.

In order to read these files into your programs, observe the following specification.

6.1. Filenames

The filenames of the real data example have the form

ECAD_{method}.txt

where *{method}* is one of *Mean*, *Var*, *LogReg* or *CoxReg*.

The filenames of the analysis for the simulated data have the form

Simulated_{method}_{mechanism}.txt

where *{method}* is one of *Mean*, *Var*, *LinReg*, *LogReg* or *CoxReg* and *{mechanism}* is one of *MCAR*, *MAR* or *MNAR*.

6.2. Rows and columns

The different rows correspond to the different subroutines. In the real data we have

1	PMM
2	MIDAStouch
3	SAMPLE
4	CART
5	RF

In the simulated data we have

1	PMM
2	SAMPLE
3	CART
4	RF

The different columns correspond to both the variables and the characteristics to be reported.

In the file *ECAD_Mean.txt* we have

1-4	Systolic blood pressure
5-8	LDL
9-12	Diabetes
13-16	Smoking behavior
17-20	Family history
1, 5, 9, 13, 17	Mean value
2, 6, 10, 14, 18	Inner standard deviation
3, 7, 11, 15, 19	Between standard deviation
4, 8, 12, 16, 20	Total standard deviation

In the files *Simulated_Mean_{mechanism}.txt* we have

1-6	Variable 5

7-12	Variable 6
13-18	Variable 7
19-24	Variable 8
25-30	Variable 9
1, 7, 13, 19, 25	Mean value
2, 8, 14, 20, 26	Standard deviation by Rubin's formula
3, 9, 15, 21, 27	Absolute bias
4, 10, 16, 22, 28	Standard deviation, simulated
5, 11, 17, 23, 29	Mean squared error
6, 12, 18, 24, 30	Coverage probability

In the file *ECAD_Var.txt* we have

1-36	Follow-up duration
37-72	Death
73-108	Age
109-144	Sex
145-180	Systolic blood pressure
181-216	LDL
217-252	Diabetes
252-288	Smoking behavior
289-324	Family history
1-4, 37-40, ..., 289-292	Follow-up duration
5-8, 41-44, ..., 293-296	Death
9-12, 45-48, ..., 297-300	Age
13-16, 49-52, ..., 301-304	Sex
17-20, 53-56, ..., 305-308	Systolic blood pressure
21-24, 57-60, ..., 309-312	LDL
25-28, 61-64, ..., 313-316	Diabetes
29-32, 65-68, ..., 317-320	Smoking behavior
33-36, 69-72, ..., 321-324	Family history
1, 5, ..., 321	Mean value
2, 6, ..., 322	Inner standard deviation
3, 7, ..., 323	Between standard deviation
4, 8, ..., 324	Total standard deviation

Notice that in order to obtain a variance, both variables have to be equal, i.e. you have to consider the columns 1-4, 41-44, 81-84, 121-124, 161-164, 201-204, 241-244, 281-284, 321-324.

In the files *Simulated_Var_{mechanism}.txt* we have

1-54	Variable 1
55-108	Variable 2
109-162	Variable 3
163-216	Variable 4
217-270	Variable 5
271-324	Variable 6
325-378	Variable 7
379-432	Variable 8
433-486	Variable 9
1-6, 55-60, ..., 433-438	Variable 1

7-12, 61-66, …, 439-444	Variable 2
13-18, 67-72, …, 445-450	Variable 3
19-24, 73-78, …, 451-456	Variable 4
25-30, 79-84, …, 457-462	Variable 5
31-36, 85-90, …, 463-468	Variable 6
37-42, 91-96, …, 469-474	Variable 7
43-48, 97-102, …, 475-480	Variable 8
49-54, 103-108, …, 481-486	Variable 9
1, 7, …, 481	Mean value
2, 8, …, 482	Standard deviation by Rubin's formula
3, 9, …, 483	Absolute bias
4, 10, …, 484	Standard deviation, simulated
5, 11, …, 485	Mean squared error
6, 12, …, 486	Coverage probability

Notice that in order to obtain a variance, both variables have to be equal, i.e. one has to choose columns 1-6, 61-66, 121-126, 181-186, 241-246, 301-306, 361-366, 421-426 and 481-486.

In the files *Simulated_LinReg_{mechanism}.txt* and *Simulated_LogReg_{mechanism}.txt* we have

1-6	Intercept
7-12	Variable 3
13-18	Variable 4
19-24	Variable 5
25-30	Variable 6
31-36	Variable 7
37-42	Variable 8
43-48	Variable 9
1, 7, …, 43	Mean value
2, 8, …, 44	Standard deviation by Rubin's formula
3, 9, …, 45	Absolute bias
4, 10, …, 46	Standard deviation, simulated
5, 11, …, 47	Mean squared error
6, 12, …, 48	Coverage probability

In the file *ECAD_LogReg.txt* we have

1-4	Intercept
5-8	Age
9-12	Sex
13-16	Systolic blood pressure
17-20	LDL
21-24	Diabetes
25-28	Smoking behavior
29-32	Family history
1, 5, …, 29	Mean value
2, 6, …, 30	Inner standard deviation
3, 7, …, 31	Between standard deviation
4, 8, …, 32	Total standard deviation

In the file *ECAD_CoxReg.txt* we have

1-4	Age
5-8	Sex
9-12	Systolic blood pressure
13-16	LDL
17-20	Diabetes
21-24	Smoking behavior
25-28	Family history
1, 5, …, 25	Mean value
2, 6, …, 26	Inner standard deviation
3, 7, …, 27	Between standard deviation
4, 8, …, 28	Total standard deviation

In the files *Simulated_CoxReg_{mechanism}.txt* we have

1-6	Variable 3
7-12	Variable 4
13-18	Variable 5
19-24	Variable 6
25-30	Variable 7
31-36	Variable 8
37-42	Variable 9
1, 7, …, 37	Mean value
2, 8, …, 38	Standard deviation by Rubin's formula
3, 9, …, 39	Absolute bias
4, 10, …, 40	Standard deviation, simulated
5, 11, …, 41	Mean squared error
6, 12, …, 42	Coverage probability
